# Quantification and determinants of the amount of respiratory syncytial virus (RSV) shed using real time PCR data from a longitudinal household study

**DOI:** 10.12688/wellcomeopenres.10284.2

**Published:** 2017-03-13

**Authors:** Miriam Wathuo, Graham F. Medley, D. James Nokes, Patrick K. Munywoki

**Affiliations:** 1 KEMRI - Wellcome Trust Research Programme, Centre for Geographic Medicine Research – Coast, Kilifi, Kenya; 2 Department of Global Health and Development, London School of Hygiene and Tropical Medicine, London, UK; 3 School of Life Sciences and SBIDER, University of Warwick, Coventry, UK; 4 Department of Nursing Sciences, Pwani University, Kilifi, Kenya

**Keywords:** RSV, viral density, shedding, household, PCR, cycle threshold

## Abstract

**Background**: A better understanding of respiratory syncytial virus (RSV) epidemiology requires realistic estimates of RSV shedding patterns, quantities shed, and identification of the related underlying factors.

**Methods**: RSV infection data arise from a cohort study of 47 households with 493 occupants, in coastal Kenya, during the 2009/2010 RSV season. Nasopharyngeal swabs were taken every 3 to 4 days and screened for RSV using a real time polymerase chain reaction (PCR) assay. The amount of virus shed was quantified by calculating the ‘area under the curve’ using the trapezoidal rule applied to rescaled PCR cycle threshold output. Multivariable linear regression was used to identify correlates of amount of virus shed.

**Results**: The median quantity of virus shed per infection episode was 29.4 (95% CI: 15.2, 54.2) log _10 _ribonucleic acid (RNA) copies * days. Young age (<1 year), presence of upper respiratory symptoms, intra-household acquisition of infection, an individual’s first infection episode in the RSV season, and having a co-infection of RSV group A and B were associated with increased amount of virus shed.

**Conclusions**: The findings provide insight into which groups of individuals have higher potential for transmission, information which may be useful in designing RSV prevention strategies.

## Introduction

Respiratory syncytial virus (RSV) is the most common viral cause of severe lower respiratory tract infection (LRTI) among infants and children under 5 years old worldwide, with the greatest burden occurring in developing countries ^1^. Most children experience an RSV infection episode by the age of two years, with peak rates of infection occurring in the first year of life ^2^. Re-infections occur throughout life ^3^ as RSV infections provide incomplete or waning immunity. There are no licensed RSV vaccines but there is heightened activity in vaccine development ^4– 8^.

Vaccine delivery strategies should be informed by detailed understanding of RSV transmission dynamics. To reduce the circulation of RSV, identifying which groups are responsible for majority of the infections is critical. Transmission potential can be estimated by a combination of mixing patterns ^9^ and virus shedding (viral density and duration). Human experimental infection with RSV reported that volunteers inoculated with a higher dose of virus (4.7 log _10_ tissue culture infectious dose (TCID) _50_ of RSV A2) were more likely to be infected than those given low doses of the inoculum (3.7 log _10_ TCID _50_ of RSV A2) ^10^, suggesting that individuals who shed more virus are likely to be more infectious. Determining quantities shed and the related underlying factors, will help in predicting the spread of infection in a population and identify key groups to target for infection control. Previous studies have used the duration of shedding to identify individuals with the greatest potential for infection spread ^11^, based on the assumption that they have a higher number of infectious contacts. Several studies report on the duration of RSV shedding with mean values ranging from 4.5 to 11.2 days ^12, 13^. In addition to variation in assay type, determinants included history of RSV infection ^12^, age, infection severity, detection of other viruses before and during the RSV infection, and presence of concurrent RSV infections in the same household ^13^. These studies and others ^14, 15^ do not account for the temporal changes in quantity of virus shed. Experimental RSV infection studies indicate that an individual infection episode begins with low viral shedding, which rises with time as the virus continues to replicate within epithelial cells and finally declines as the infection clears ^16^.

The current analysis aims to include temporal changes in viral shedding in estimating the total amount of virus shed during an individual RSV infection episode. Relative to the duration of shedding, this may provide an improved correlate of infectiousness and help in identifying the key factors influencing RSV shedding patterns. Such data are informative in formulating vaccine product profiles and designing prevention strategies for RSV.

## Methods

### Data

The RSV infection data arise from an intensively followed cohort of 47 households with 493 occupants in rural coastal Kenya. The details of the study have been described elsewhere ^13, 17, 18^. In summary, throughout an RSV season spanning 26 weeks (December 2009–June 2010), nasopharyngeal swabs (NPS) were collected by trained field assistants every 3–4 days, irrespective of symptoms, from 47 RSV naïve infants and their household members. Households were selected through the Kilifi Health and Demographic Surveillance System (KHDSS) and local community health workers and were considered eligible if they had a child born after 1 ^st^ April 2009. The infants were assumed to be RSV naïve because they were born after the 2008/2009 RSV season. A total of 16928 NPS collections were tested for RSV (groups A and B) and other prevalent respiratory viruses (adenoviruses, rhinoviruses and human coronaviruses (NL63, 229E and OC43)) using multiplex real time polymerase chain reaction (PCR) assay as previously described ^19^. As PCR cycles proceed the quantity of product detected rises and at some point passes through a user assigned threshold for detection (set at the point of observable exponential rise in product), known as the cycle threshold (Ct). The earlier (lower) the Ct value the higher the starting concentration of target sequence (equated with viral density). As a cut-off, samples with Ct values of 35.0 and below were considered positive. An RSV infection episode was defined as the period within which an individual provided specimens which were PCR positive for the same infecting RSV group with no more than 14 days separating any two positive samples. A household outbreak was defined as a period in which a household experienced more than one individual infection episode, without there being more than 14 days between any two infection episodes. A primary/index case was a person first identified to have an RSV infection leading to a household outbreak. A first episode was the first episode in that season that an individual had while a subsequent episode was a second or third episode that a person may have had during the follow up. Virus test results, recorded in Ct values, were converted to log _10_ ribonucleic acid (RNA) copy numbers (direct measure of viral density) to enable plotting of the time – concentration curve. The equation *y = -3.308x + 42.9* was used to convert Ct values (y), to their log _10_ RNA equivalents (x), as a way of quantifying RNA ^20^. The converted values are hereafter referred to as viral densities. Amount of virus shed during an infection episode was estimated by calculating the area under the time-concentration curve (AUC) using the trapezoidal rule ^21^, with units of copies * days. The peak viral density for each episode was identified as the highest measured viral density in an infection episode.

### Calculation of area under curve

**Figure 1.**
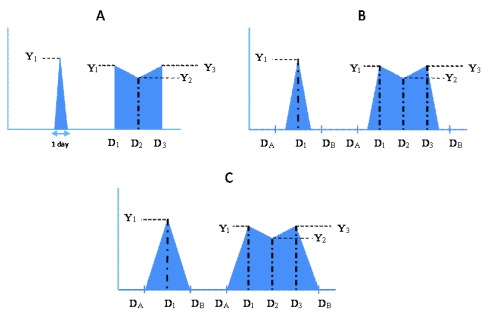
Schema depicting the patterns of virus shedding for three possible scenarios, **A**, **B** and **C**, graphically representing the minimum, midpoint and maximum AUC calculations, respectively, defined in the Methods text. For each scenario two example episodes are shown, the first with only one virus positive sample, and the second with three virus positive samples. Y _i_ – Log viral density values; D _A_ – Day of last negative sample before start of episode; D _B_ – Day of first negative sample after end of episode; D _1_ – Day of first positive sample; D _2_ – Day of second positive sample; D _3_ – Day of third positive sample.

For every episode, calculation of the area under the curve involved plotting a time concentration curve of the different log viral densities over the duration of shedding of the episode ( Figure 1). Three scenarios were explored which take account of uncertainty arising from the sampling intervals, estimating a minimum, midpoint and maximum AUC, for which calculations are described below, aided by illustrations in Figure 1A – C. For each scenario, two examples are described: the first with one positive observation, and the second with three positive observations ( Figure 1). This analogy can be extended to episodes with a different number of positive observations. The number of positive observations in an episode ranged from 1 to 11.

***a) Minimum area under curve.*** The minimum AUC estimate assumed that an individual started shedding on the day of the first positive sample in an infection episode, and stopped shedding on the day of the last positive sample of the episode, in the form shown in Figure 1A, with viral density Y _1_(log _10_ RNA copies), and hence calculated as,

AUC = 0.5 * Y _1_ * 1 day.

If an episode had two or more consecutive positive observations, AUC was calculated by including all days in which they were assumed to be shedding. Thus, for an example episode with three positive observations, with viral density Y _i_, i=1,3, respectively, then:

AUC = (0.5 *((D _2_– D _1_) +1) * (Y _1_+ Y _2_)) + (0.5 * (D _3_– D _2_) * (Y _2_+ Y _3_))…………….….. (i).

Note the addition of one day to the calculation such that shedding on day 1 is included. Days with zero Ct values in between samples with positive Ct values were taken to have zero viral density, after conversion of the Ct values to log _10_ viral RNA copies. Therefore, AUC was calculated as in (i) above including both zero and non-zero observations. For left and right censored episodes, calculation of the amount of virus remained as in (i) above.

***b) Midpoint area under curve.*** The midpoint AUC estimate assumed that an individual began shedding midway between the day of the first positive sample of an infection episode and the day of the last negative sample before the start of the episode. An individual was assumed to have stopped shedding midway between the day of the last positive sample of an episode and the day of the first negative sample after the episode ( Figure 1B). 

Hence to calculate the AUC for an episode with one positive observation:

Let the midpoint days be: x = (D _A_ + D _1_)/2, and y = (D _B_ + D _1_)/2, where D _A_, D _B_ and D _1_ are, respectively, the day of the last negative sample before the start of the episode; day of the first negative sample after the end of the episode; day of the first positive sample, hence

AUC = 0.5 *((y – x) + 1) * Y _1._

To calculate the AUC for an episode with three positive observations:

Let the midpoint days be: x = (D _A_ + D _1_)/2, and y = (D _B_ + D _3_)/2, where D _2_ and D _3_ are the days of the second and third positive samples, respectively, so

AUC = (0.5 * Y _1_ * ((D _1_- x) +1)) + (0.5 * (D _2_ – D _1_) * (Y _1_ + Y _2_)) + (0.5 * (D _3_– D _2_) * (Y _2_ + Y _3_)) + (0.5 * Y _3_ * (y - D _3_)) …………. (ii).

Days with zero Ct values in between samples with positive Ct values were taken to have a Ct value of 40 (the Ct value translating to the lowest viral load), then converted to viral density along with other Ct values in the episode, and the AUC calculated as in (ii) above.

For left and right censored episodes, with unknown D _A_ and D _B_, respectively, shedding was assumed to start 1.85 days (i.e. half the mean sampling interval) before the first observed day of shedding, D _1_, and to finish 1.85 days after the last positive sample (D _3_ in the example of (ii) above). Hence, in (ii) above, (D _1_-x) and (y-D _3_) were replaced with the value 1.85 days, for left and right censored data, respectively.

***Maximum area under curve.*** To calculate the maximum AUC, individuals were assumed to have begun shedding immediately after the last negative sample before the start of an episode, and stopped shedding immediately before the first negative sample after the episode ( Figure 1C). The viral densities for the days when individuals were considered to have begun and stopped shedding were assumed to be zero. Zero Ct values within an infection episode were assigned viral densities equal to the average recorded for the first positive sample before and firstpositive sample after the zero Ct samples.

Hence, to calculate the AUC for an episode with one positive observation:

AUC = 0.5 *(D _B_ – D _A_) * Y _1_,

and to calculate the AUC for an episode with three positive observations:

AUC = (0.5 * Y _1_ * (D _1_- D _A_)) + (0.5 * (D _2_– D _1_) * (Y _1_+ Y _2_)) + (0.5 * (D _3_– D _2_) * (Y _2_ + Y _3_)) + (0.5 * Y _3_ * (D _B_ - D _3_)) ……………………(iii).

Left and right censored episodes were treated similarly to that for midpoint AUC, but assuming shedding began and ended 3.7 days (i.e. the mean sampling interval) before the first positive sample of an episode and after the last positive sample of an episode. Hence in (iii) above, (D _1_-D _A_) and (D _B_-D _3_) were replaced with the value 3.7 days, for left and right censored data, respectively.

### Statistical analysis

Data were analysed using Stata version 13.1 (StataCorp. College Station, TX: StataCorp LP. RRID:SCR_012763) and RStudio version 0.99.489 (RStudio Team. RStudio, Inc., Boston, MA. RRID:SCR_000432). The differences in the distribution of amount of virus by various characteristics was tested using the Wilcoxon rank-sum test and Kruskal Wallis test as appropriate. Spearman's rank correlation coefficient was used to find the association between the different measures of viral quantity calculated. Linear regression was used to identify the main factors associated with the amount of virus shed, i.e. AUC. Possible covariates were chosen from the dataset by selecting those that could plausibly be associated with AUC. A univariable analysis was carried out to identify factors associated with AUC. All variables with a p-value of <0.1 in the univariable analysis were included in the multivariable regression analysis. The final multivariable regression model was developed by a backward elimination procedure, removing variables with a p-value >0.05 in each step using likelihood ratio tests. Risk factors were removed in descending order of strength of association determined from the multivariable analysis. Two interactions were tested in the multivariable model. An interaction between age and symptom status was tested to determine if the effect of age on the amount of virus shed varied by symptom status. An interaction between age and primary/secondary case status was also tested because it was suspected that the effect of age on the amount of virus shed could vary by primary/secondary case status. We chose to adjust for sex *a priori* because it was considered an important risk factor to acute respiratory infections ^22^

### Ethics

The household study was approved by the KEMRI Ethical Review Committee in Kenya, SSC No. 1651, and the Biomedical Research Ethics Committee at the University of Warwick in the United Kingdom. Data was anonymised by using unique identification numbers for each participant and household.

### Consent

Written informed consent was obtained from all participants and/or their parents or guardians.

## Results

### Infection episodes

The mean (SD) sampling interval was 3.7 (2.3) days. The median number of swabs collected for an individual was 41, with the minimum being 1 swab and the maximum 48 swabs. There were 537 (3.2%) samples from 179 individuals that were positive for RSV. RSV group A only, group B only, and group A/B co-infections were detected in 231 (1.5%), 287 (1.8%), and 19 (0.1%) NPS collections, respectively ( Table 1). From the 179 infected individuals, a total of 208 infection episodes were observed during the six-month study period; 180 were fully observed episodes while 13 and 17 were left and right censored episodes respectively. Two episodes were both left and right censored. Eighty three (39.9%), 111 (53.4%), and 14 (6.7%) episodes were associated with RSV group A, group B and a co-infection respectively. One hundred and fifty two (84.9%) individuals had one episode, while 25 (14.0%) and 2 (1.1%) had two and three episodes respectively ( Table 1). In addition, 24 (60.0%) households had one outbreak, while 5 (12.5%) had two outbreaks.

**Table 1.** Distribution of RSV infection episodes among individuals during the 2009/2010 RSV season in a household cohort, coastal Kenya.

Characteristic	Categories	Individuals in study (n=493)	Individuals infected (n=179)		Infection episodes (n=208)		Total AUC ^a^ (n=7875.47)	
		n	n	%	n	%	n	%
Age at infection	<1y	55	31	17.3	35	16.8	2424.1	30.7
	1-<5y	82	41	22.9	51	24.5	2011.8	25.5
	5-<15y	165	64	35.8	73	35.1	2241.7	28.4
	15-<40y	147	35	19.6	40	19.2	854.0	10.8
	> = 40y	44	8	4.5	9	4.3	366.9	4.6
Sex	Female	272	96	53.6	118	56.7	4392.4	55.6
	Male	221	83	46.4	90	43.3	3506.2	44.4
Symptomatic	No	87	71	39.7	90	43.3	2145.2	27.2
	Yes	406	108	60.3	118	56.7	5753.3	72.8
Participant in school ^b^	No	259	101	56.4	119	57.2	5184.2	65.6
	Yes	204	78	43.6	89	42.8	2714.4	34.4
Living with smoker in household	No	389	155	86.6	181	87	7081.2	89.7
	Yes	104	24	13.4	27	13	817.3	10.3
Household size	< = 5	37	22	12.3	25	12	992.6	12.6
	6 to 10	180	52	29.1	61	29.3	2605.3	33.0
	11 to 15	101	35	19.6	40	19.2	1360.5	17.2
	>15	175	70	39.1	82	39.4	2940.1	37.2
Infection with other viruses	No	43	83	46.4	110	52.9	5054.6	64.0
	Yes	450	96	53.6	98	47.1	2844.0	36.0
In household with outbreak	No	162	14	7.8	192	92.3	7460.6	94.5
	Yes	331	165	92.2	16	7.7	438.0	5.5
Infecting RSV group ^c^	RSV A		66	36.9	83	39.9	2838.1	35.9
	RSV B		91	50.8	111	53.4	4170.0	52.8
	RSV A & B		22	12.3	14	6.7	890.5	11.3
Order of the episode in individual	First		152	84.9	179	86.1	7130.3	90.3
	Subsequent		27	15.1	29	13.9	768.3	9.7
Introducer of RSV infection in household	Primary case		66	36.9	70	33.7	2372.7	30.0
	Others		113	63.1	138	66.4	5525.9	70.0

^a^Total amount of virus shed (log _10_ RNA copies * days). ^b^Includes some participants in the age groups 1-<5 years, 5-<15 years, and 15-<40 years only. ^c^Individuals could appear in one or more categories of this variable.

### Overall amount of RSV shed

The amount of virus shed was presented in logarithmic form because the untransformed values were skewed to the right, and the residuals of the linear regression analysis using the untransformed outcome did not meet the normality assumption. Using log transformed values also helped to reduce the variance. The mean (variance) for the untransformed and log-transformed values was 3.73×10 ^7^ (1.34×10 ^8^) and 6.07 (1.51), respectively.

**Figure 2.**
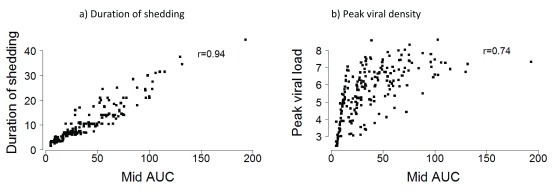
Scatter plots showing the correlation between ( **a**) duration of shedding (days) and ( **b**) peak viral density (log _10_ RNA copies) and midpoint AUC (log _10_ RNA copies*days) estimates.

The median (interquartile range, IQR) of the amount of virus shed estimated by the minimum, midpoint and maximum AUC approaches was 31.1 (15.4, 57.9), 41.7 (24.3, 68.0), and 50.7 (31.0, 79.6) log _10_ RNA copies per infection episode, respectively. The minimum and maximum AUC estimates were strongly correlated with the midpoint AUC estimates (r=0.97 P < 0.001 and r=0.96 P < 0.001, respectively) ( Supplementary Material Figure S1). The alternative measures of RSV shedding, (shedding duration and peak viral density), were also strongly correlated to the midpoint AUC estimates; 0.94 (P < 0.001) and 0.74 (P < 0.001) ( Figure 2). Note, however, that within a narrow range of AUC the range of peak virus can be wide. The distribution of the amount of RSV shed by the various characteristics explored was similar regardless of the estimation method (i.e. minimum, midpoint, or maximum AUC) (See Supplementary Material Table S1) and hereafter only the midpoint estimates are reported. RSV viral loads for both RSV A and RSV B seemed to be low at the beginning of the RSV season, but they later increased well into the season. However, the peak viral loads reached during the season for both RSV A and RSV B seemed to be similar (see Supplementary Material Figure S2).

### Amount of RSV shed by various characteristics

**Figure 3.**
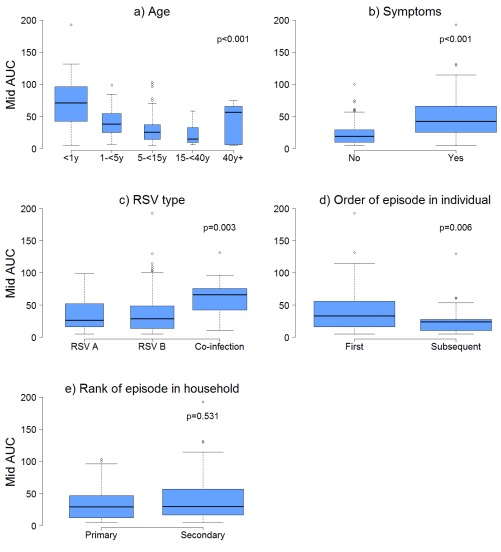
Box and whisker plots showing the distribution of amount of virus shed (AUC) using the midpoint estimates by **a**) age **b**) symptoms **c**) RSV type **d**) order of episode in individual, and **e**) primary or secondary episode in household. The width of a box relative to other boxes in the same plot is directly proportional to the mean duration of shedding in each category of that plot.

**Figure 4.**
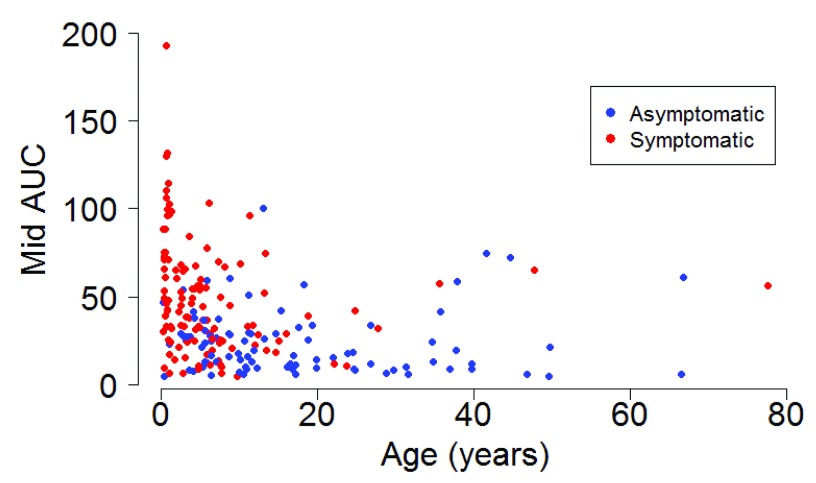
Scatter plot showing the relationship between midpoint AUC and age stratified by symptom status.

**Figure 5.**
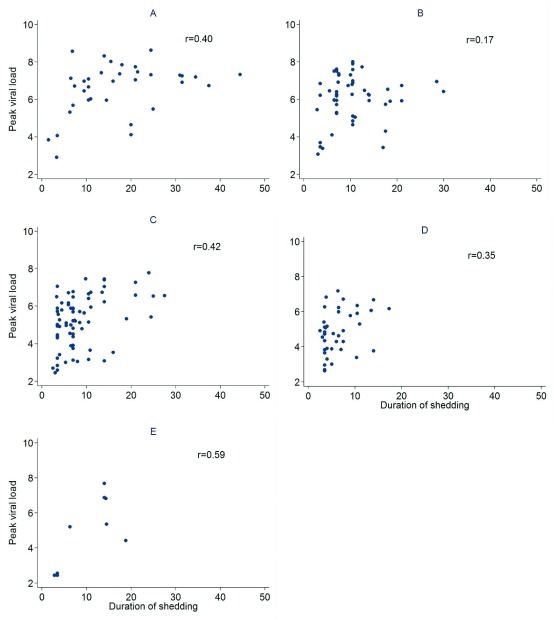
Scatter plots showing the relationship between duration of shedding (days) and peak viral density (log _10_ RNA copies) for individuals aged **a**) less than 1 year old, **b**) 1 to 4 years, **c**) 5 to 14 years, **d**) 15 to 39 years, and **e**) 40 years and above.

The median (IQR) amount of virus shed was 71.0 (42.3, 96.7), 37.7 (24.6, 54.4), 25.0 (14.1, 36.7), 14.6 (9.4, 32.4), and 56.3 (5.7, 65.3) log _10_ RNA copies for individuals aged <1y, 1-<5y, 5-<15y, 15-<40y and >=40y respectively ( Figure 3a) based on the midpoint approach, and there was strong evidence of a difference in distribution of AUC in different age groups (P = 0.001). Infection episodes associated with symptoms had a higher median (IQR) amount of virus shed than those without symptoms (42.5 (25.1, 66.0) vs 19.0 (9.8, 29.0) log _10_ RNA copies; P < 0.001) ( Figure 3b). Most symptomatic episodes were experienced by younger individuals (<20 years), while older individuals above 20 years mostly had asymptomatic episodes ( Figure 4). Episodes associated with RSV A only, RSV B only, and RSV group A/B co-infection had a median amount of virus of 26.4 (15.1, 52.4), 28.8 (13.2, 48.9), and 66.1 (42.1, 75.5) log _10_ RNA copies respectively (P = 0.003) ( Figure 3c). Co-infection (associated with adenoviruses, rhinovirus, or coronaviruses) episodes had a median amount of virus of 38.7 (19.6, 65.3), while episodes not associated with any other infection had a median of 25.0 (11.1, 37.0) log _10_ RNA copies, and there was strong evidence (P = 0.003) of a difference in the distribution of AUC in these two groups of episodes. Episodes that occurred during a household outbreak had a median (IQR) amount of virus of 31.6 (15.9, 54.8) log _10_ RNA copies, compared to a median (IQR) of 20.3 (10.2, 35.4) log _10_ RNA copies for episodes that were not associated with household outbreaks, though there was weak evidence of a difference in medians (see Supplementary Material Table S1). Infants had the highest peak viral density, and longest duration of shedding ( Figure 5). Lowering the threshold below 35 Ct did not greatly change the results but reduced the total number of infections (see Supplementary Material Figure S3).

### Factors associated with the amount of virus shed

The final multivariable regression model identified age, symptom status, RSV infecting group, order of the episode in an individual, and being a primary/secondary case in the household as the main factors associated with the amount of virus shed. The effect of age on the amount of virus shed seemed to vary with primary/secondary case status ( Table 2). The results reported here are, therefore, adjusted for the above covariates. The difference in mean amount of virus in secondary case episodes for age groups 1-<5y, 5-<15y, 15-<40y, and >=40y when compared to infants <1y was; -41.6 (95% CI: -53.7, -29.6), -43.1 (-55.5, -30.7), -50.6 (95% CI: -64.9, -36.3), and -29.4 (95% CI: -50.8, -8.1) log _10_ RNA copies respectively (P < 0.001). There was no evidence (p=0.594) of a difference in amount of virus by age for primary cases. There was an increase in mean amount of virus for symptomatic episodes compared to asymptomatic episodes by 14.1 (95% CI: 6.3, 21.9) log _10_ RNA copies, and strong evidence of a difference in means (P < 0.001). Mean amount of virus shed for subsequent episodes of individuals in the study RSV season compared to first infections was -10.4 (95% CI: -20.0, -0.8) log _10_ RNA copies (P = 0.03). The difference in mean amount of virus for episodes of RSV B only and RSV A/B co-infections when compared to episodes of RSV group A only was 3.7 (95% CI: -3.1, 10.5) log _10_ RNA copies, and 21.3 (95% CI: 7.3, 35.3) log _10_ RNA copies (P = 0.012) respectively ( Table 2). For all age groups, there was no evidence of a difference in amount of virus between primary and secondary episodes. There was no evidence that the effect of age on amount of virus varied by symptom status.

**Table 2.** Factors influencing amount of virus shed in rural coastal Kenya during the 2009/2010 RSV season: Multiple linear regression analysis.

Factors	Categories	Beta ^a^ (log _10_ RNA copies)	95% CI	P-value
**Primary cases:** Age in years	<1y	Ref *		
	1-<5y	-0.21	-18.4, 17.9	0.594
	5-<15y	-7.3	-23.0, 8.4	
	15-<40y	-12.7	-30.7, 5.2	
	> = 40y	-9.4	-39.3, 20.5	
**Secondary cases:** Age in years	<1y	Ref *		
	1-<5y	-41.6	-53.7, -29.6	<0.001
	15-<15y	-43.1	-55.5, -30.7	
	15-<40y	-50.6	-64.9, -36.3	
	>=40y	-29.4	-50.8, -8.1	
**Symptomatic**	No	Ref *		
	Yes	14.1	6.3, 21.9	<0.001
**Order of infection** **in individual**	First	Ref *		
	Subsequent	-10.4	-20.0, -0.8	0.03
**RSV group**	RSV A	Ref *		0.012
	RSV B	3.7	-3.1, 10.5	
	Co-infection	21.3	7.3, 35.3	
**Sex**	Female	Ref *		0.287
	Male	-4.0	-10.2, 3.0	

^a^Difference in mean AUC in comparison to reference category using midpoint estimates. *Reference category.

## Discussion

We report a detailed analysis of RSV shedding patterns in which the amount of virus shed during the course of an infection was quantified, and factors associated with the quantity shed were identified. Young age at infection, presence of respiratory symptoms, intra-household acquisition of infection, being a first infection episode in the season for an individual, and having a co-infection were associated with increased amounts of RSV shed. The majority of virus available for transmission during an RSV epidemic appears to arise from individuals in their first year of life and therefore undergoing their first RSV infection episode (since all were born after the preceding RSV epidemic). Household size did not influence the per person amount of virus shed, and neither did the presence of a smoker in the household. In this rural community, indoor smoke from solid fuels (mainly firewood and charcoal) was ubiquitous and likely to conceal any specific effects of cigarette smoke. In addition, we only had 18 smokers out of the 493 participants. So, relatively few people lived with smokers. Also, we did not collect data on contact patterns of household members with the smokers or if the smoker did smoke in the house or outside which could affect the level of exposure to smoke within the households. The apparent effect of school going status on amount of virus shed was confounded by age, as school was attended by young individuals, who in turn shed more virus. RSV viral densities appeared to be low for most individuals in the beginning of the season but increased well into the season. This was a reflection of the occurrence of the RSV epidemic. Viral densities for RSV B were first to increase followed by viral densities for RSV A. RSV B infections spread to more individuals probably due to earlier onset by chance compared to RSV A infections.

The finding that 90.3% of virus shed was from individuals experiencing their first episode in the RSV season indicates that first infections are most important in virus transmission compared to subsequent infections ( Table 1). Modelling studies have been unable to determine to what extent reinfections contribute to transmission ^11, 23^, which has a big influence on predictions of the effect of vaccination on transmission dynamics ^11^. Whilst these results do not include the opportunities to transmit, which will be generally higher for school-going children undergoing their second or third infection ^9^, they suggest that people with their first episode in the season are the most likely to transmit infection when they contact susceptible individuals. Peak viral density and duration of infection episodes were highly correlated with the amount of virus shed. Infants had the highest peak viral density, and longest duration of shedding ( Figure 5).

Infants shed the highest median amount of virus followed by individuals aged 40 and above. The adults (15 to 39 years) shed the lowest amounts ( Figure 3a). Acquired immunity (following exposure to RSV) and physiological development of the airways with age have been linked with reduced risk of severe RSV disease ^12, 13^. We speculate that elderly individuals are more prone to infection because of their deteriorating immune response which could explain the higher amount of virus in individuals over 40 years, compared to other adults. However, this study cannot discriminate between the two factors of young age and a first infection as the dominant factors associated with shedding (infants all had first infections, and we do not know the history of previous infection in all other ages). Individuals with a co-infection of RSV group A and B shed twice the amount of virus compared to those infected with either RSV A or B only, suggesting that the physiological infection processes for two viruses might be independent (rather than competitive or synergistic). Similar patterns of shedding were reported based on shedding duration estimates using this dataset ^13^ and others ^12, 15^. These findings can provide a hint to pathogenesis of RSV disease ^6^. A recent experimental study has reported disease severity to be closely related to viral load ^16^ even though another shows contrasting observations on disease severity and viral load link ^10^. Our study showed a strong positive association between virus shedding and presence of respiratory symptoms. Symptomatic individualso shed higher amounts of virus than asymptomatic individuals irrespective of age. Overall those who were both young (<5 years) and symptomatic contributed to the highest amount of virus shed (51.8%). Using amount of virus as a correlate of infectiousness, these two factors are associated with virus spread within the community and in families and thus have direct implications in design and delivery of transmission-blocking interventions such as vaccines.

This analysis had some limitations. First, the nasal samples were collected with intervals of 3 to 4 days hence a complete profile of the viral density changes over time could not be captured. This has implications on the accuracy of our AUC estimates. However we minimize this by using a conservative approach providing three possible estimates i.e. minimum, midpoint and maximum estimates as detailed in the Supplementary Material. Furthermore, the three measures were strongly correlated and their variations by various characteristics such as age and symptoms, were comparable. Second, Ct values were converted to RNA copies using published associations ^20^ hence providing only a relative measure of virus density in the sample taken rather than absolute values of the amount of virus shed. Using the inverse of the Ct values as a measure of viral load did not change the distribution of AUC by various characteristics or any conclusions made from the results. There are no published studies using our approach in a community setting to facilitate comparison. Our approach takes into account both changing viral load and the duration of shedding making it more robust. However, its accuracy also relies on user selected thresholds for measures of viral load, and this could result in some differences in findings across studies. A quantitative PCR would be preferable. Third, there is inherent variability in estimated viral load due to variation (both biological and methodological) in the amount of virus collected using a nasopharyngeal swab. Deep NPS collections might have a higher density than perinasal swabs but events prior to sampling, e.g. sneezing, might introduce variations in sample quality. However this is likely to be non-systematic hence only conservatively affects our final conclusions. Finally, the assumption is made that virus density as measured by PCR is a measure of infectious virus. The most likely limitation of this assumption is over-estimating the duration of and peak shedding of viable virus. Generally, the relationship between the measures of viral shedding and infectiousness to other people are unknown. In the current analysis we took logarithms of estimated viral densities, under the general assumption that the risk of transmission will saturate at higher viral shedding. We then integrated over this logarithmic measure to obtain a measure of total infectiousness. An alternative would be to have integrated over the untransformed viral density, and then taken logarithms prior to statistical analysis. This approach did not give substantially different results. Estimation of the functional relationship between viral shedding and transmission is being considered in on-going analysis of the epidemics observed within households.

In conclusion, long shedders tend to shed more virus and are likely to be more infectious. However, it is also evident that individuals with similar durations of shedding may shed different amounts of virus. Individuals with a combination of high viral density and long shedding durations are likely to shed the most virus. This improves on existing literature that measures infectiousness using the duration of shedding only. The groups that are most likely to spread infection are individuals with a first infection episode, the symptomatic, and most predominantly, the young, which has implications on which groups to target for vaccination in RSV prevention strategies.

## Data availability

The dataset used in this study, together with associated analysis code (.do), graphics code (.R), variable codebook (.pdf), and summary file (.txt) are available from Harvard Dataverse: doi: 10.7910/DVN/MOTEJH ^24^. The dataset has restricted access, retaining Personal Identifiable Information (specifically dates) for analysis duplication. Please request access as instructed in the Readme.txt file. Other files are open access.

## Supplementary material

This file provides (a) Scatter plots showing the association between minimum, midpoint and maximum AUC ( **Figure S1**), (b) graphical presentation of the relationship between amount of virus and duration of shedding for different thresholds of positivity ( **Figure S2**), and temporal patterns of virus load ( **Figure S3**), and (c) descriptive analysis tables for virus shedding ( **Table S1** and **Table S2**).

## References

[1] Nair H Nokes DJ Gessner BD: Global burden of acute lower respiratory infections due to respiratory syncytial virus in young children: a systematic review and meta-analysis. *Lancet.* 2010;375(9725):1545–1555. 10.1016/S0140-6736(10)60206-1 20399493 PMC2864404

[2] Glezen WP Taber LH Frank AL: Risk of primary infection and reinfection with respiratory syncytial virus. *Am J Dis Child.* 1986;140(6):543–6. 10.1001/archpedi.1986.02140200053026 3706232

[3] Henderson FW Collier AM Clyde WA Jr: Respiratory-syncytial-virus infections, reinfections and immunity. A prospective, longitudinal study in young children. *N Engl J Med.* 1979;300(10):530–4. 10.1056/NEJM197903083001004 763253

[4] Modjarrad K Giersing B Kaslow DC: WHO consultation on Respiratory Syncytial Virus Vaccine Development Report from a World Health Organization Meeting held on 23–24 March 2015. *Vaccine.* 2016;34(2):190–7. 10.1016/j.vaccine.2015.05.093 26100926 PMC6858870

[5] Karron RA Wright PF Belshe RB: Identification of a recombinant live attenuated respiratory syncytial virus vaccine candidate that is highly attenuated in infants. *J Infect Dis.* 2005;191(7):1093–104. 10.1086/427813 15747245

[6] Wright PF Karron RA Belshe RB: The absence of enhanced disease with wild type respiratory syncytial virus infection occurring after receipt of live, attenuated, respiratory syncytial virus vaccines. *Vaccine.* 2007;25(42):7372–7378. 10.1016/j.vaccine.2007.08.014 17868959 PMC2760483

[7] Anderson LJ Dormitzer PR Nokes DJ: Strategic priorities for respiratory syncytial virus (RSV) vaccine development. *Vaccine.* 2013;31(Suppl 2):B209–15. 10.1016/j.vaccine.2012.11.106 23598484 PMC3919153

[8] Guvenel AK Chiu C Openshaw PJ: Current concepts and progress in RSV vaccine development. *Expert Rev Vaccines.* 2014;13(3):333–44. 10.1586/14760584.2014.878653 24405366 PMC7612829

[9] Kiti MC Kinyanjui TM Koech DC: Quantifying age-related rates of social contact using diaries in a rural coastal population of kenya. *PLoS One.* 2014;9(8):e104786. 10.1371/journal.pone.0104786 25127257 PMC4134222

[10] Lee FE Walsh EE Falsey AR: Experimental infection of humans with A2 respiratory syncytial virus. *Antiviral Res.* 2004;63(3):191–196. 10.1016/j.antiviral.2004.04.005 15451187

[11] Kinyanjui TM House TA Kiti MC: Vaccine Induced Herd Immunity for Control of Respiratory Syncytial Virus Disease in a Low-Income Country Setting. *PLoS One.* 2015;10(9):e0138018. 10.1371/journal.pone.0138018 26390032 PMC4577090

[12] Okiro EA White LJ Ngama M: Duration of shedding of respiratory syncytial virus in a community study of Kenyan children. *BMC Infect Dis.* 2010;10:15. 10.1186/1471-2334-10-15 20096106 PMC2822777

[13] Munywoki PK Koech DC Agoti CN: Influence of age, severity of infection, and co-infection on the duration of respiratory syncytial virus (RSV) shedding. *Epidemiol Infect.* 2015;143(4):804–12. 10.1017/S0950268814001393 24901443 PMC4411640

[14] Hall CB Geiman JM Biggar R: Respiratory syncytial virus infections within families. *N Engl J Med.*[electronic article].1976;294(8):414–9. 10.1056/NEJM197602192940803 173995

[15] Frank AL Taber LH Wells CR: Patterns of shedding of myxoviruses and paramyxoviruses in children. *J Infect Dis.*[electronic article].1981;144(5):433–41. 10.1093/infdis/144.5.433 6273473

[16] DeVincenzo JP Wilkinson T Vaishnaw A: Viral load drives disease in humans experimentally infected with respiratory syncytial virus. *Am J Respir Crit Care Med.* 2010;182(10):1305–1314. 10.1164/rccm.201002-0221OC 20622030 PMC3001267

[17] Munywoki PK Koech DC Agoti CN: Frequent Asymptomatic Respiratory Syncytial Virus Infections During an Epidemic in a Rural Kenyan Household Cohort. *J Infect Dis.* 2015;212(11):1711–8. 10.1093/infdis/jiv263 25941331 PMC4633757

[18] Munywoki PK Koech DC Agoti CN: The source of respiratory syncytial virus infection in infants: a household cohort study in rural Kenya. *J Infect Dis.* 2014;209(11):1685–92. 10.1093/infdis/jit828 24367040 PMC4017365

[19] Hammitt LL Kazungu S Welch S: Added value of an oropharyngeal swab in detection of viruses in children hospitalized with lower respiratory tract infection. *J Clin Microbiol.* 2011;49(6):2318–2320. 10.1128/JCM.02605-10 21490188 PMC3122752

[20] Nolan T Hands RE Bustin SA: Quantification of mRNA using real-time RT-PCR. *Nat Protoc.* 2006;1(3):1559–1582. 10.1038/nprot.2006.236 17406449

[21] Kreide D Lahr D: Trapezoid Rule and Simpson’s Rule.2010 Reference Source

[22] Simoes EA: Environmental and demographic risk factors for respiratory syncytial virus lower respiratory tract disease. *J Pediatr.* 2003;143(5 Suppl):S118–S126. 10.1067/S0022-3476(03)00511-0 14615710

[23] White LJ Waris M Cane PA: The transmission dynamics of groups A and B human respiratory syncytial virus (hRSV) in England & Wales and Finland: seasonality and cross-protection. *Epidemiol Infect.* 2005;133(2):279–89. 10.1017/S0950268804003450 15816153 PMC2870247

[24] Munywoki PK Nokes DJ: Replication Data for: Quantification and determinants of the amount of respiratory syncytial virus (RSV) shed using real time PCR data from a longitudinal household study. *Harvard Dataverse, V5.* 2016 Data Source 10.12688/wellcomeopenres.10284.2 PMC5218551 28066826

