# Supplementary material for: Quantification and determinants of the amount of respiratory syncytial virus (RSV) shed using real time PCR data from a longitudinal household study
Source: Wellcome Open Res. 2017 Mar 13;1:27. Originally published 2016 Dec 14. [Version 2] doi: 10.12688/wellcomeopenres.10284.2 (PMC5218551; doi:10.12688/wellcomeopenres.10284.2)
Supplement: Supplementary file 1 [file wellcomeopenres-1-11638-s0000.tgz › 667bb7b5-c802-4f40-9038-37a539c6ee9f.docx]

**Quantification and determinants of the amount of respiratory syncytical virus (RSV) shed using real time PCR data from a longitudinal household study.**

**Supplementary material**


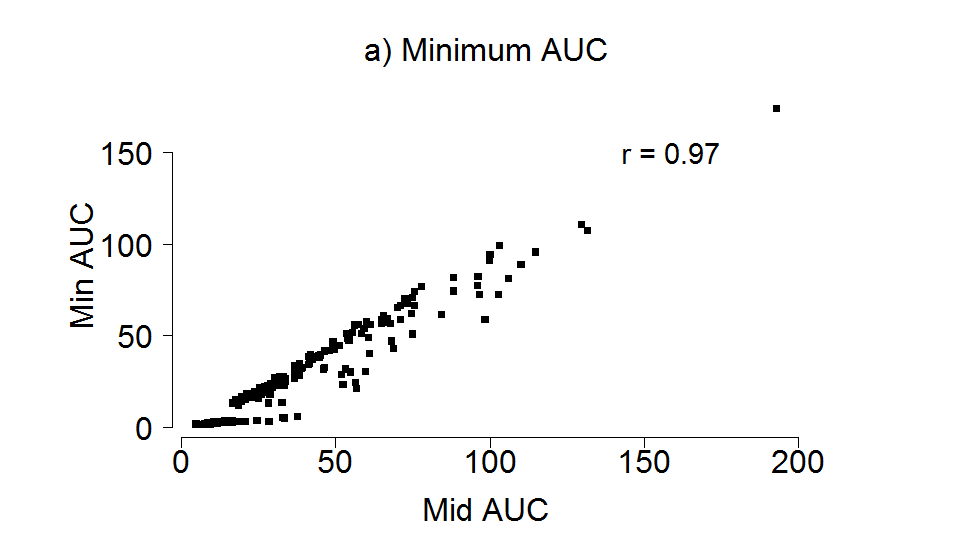

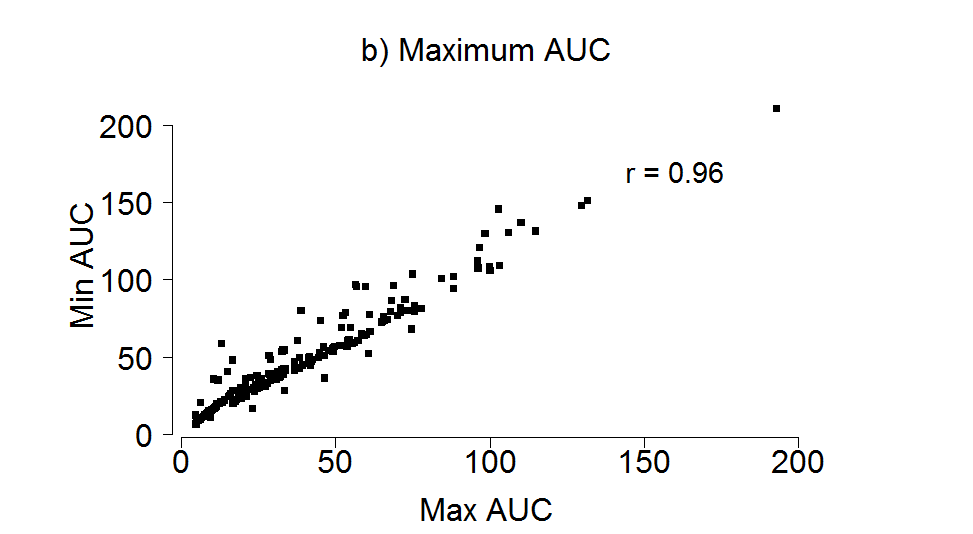


*Figure S1: Scatter plots showing relationship of minimum and maximum AUC to midpoint AUC.*


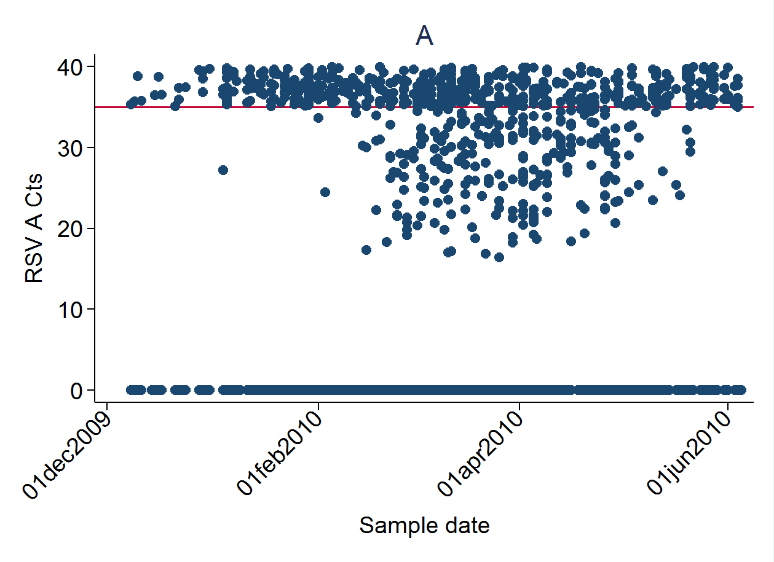

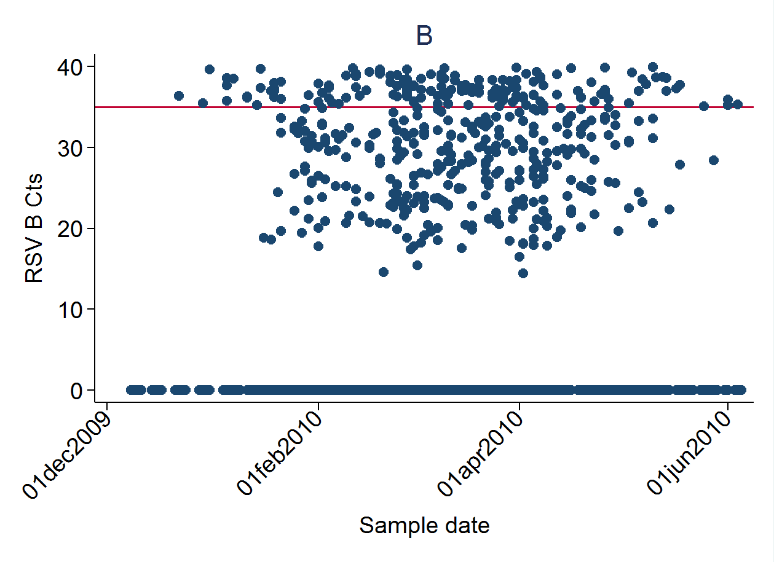

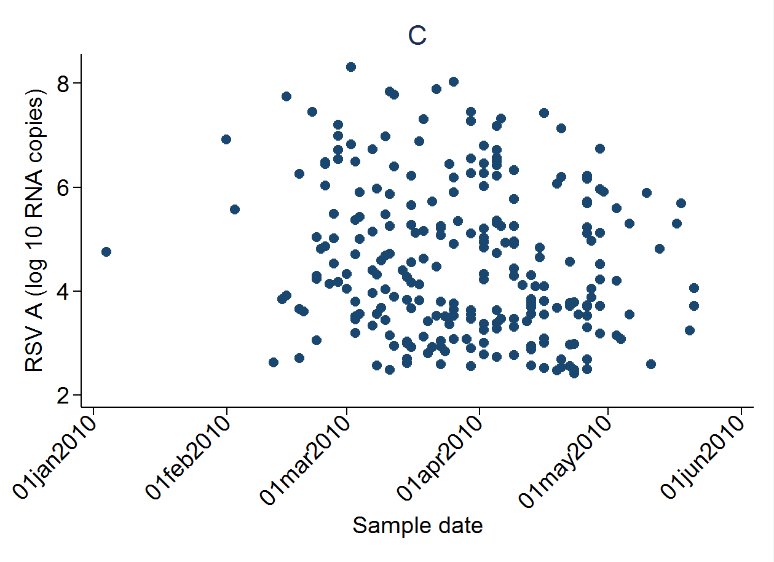

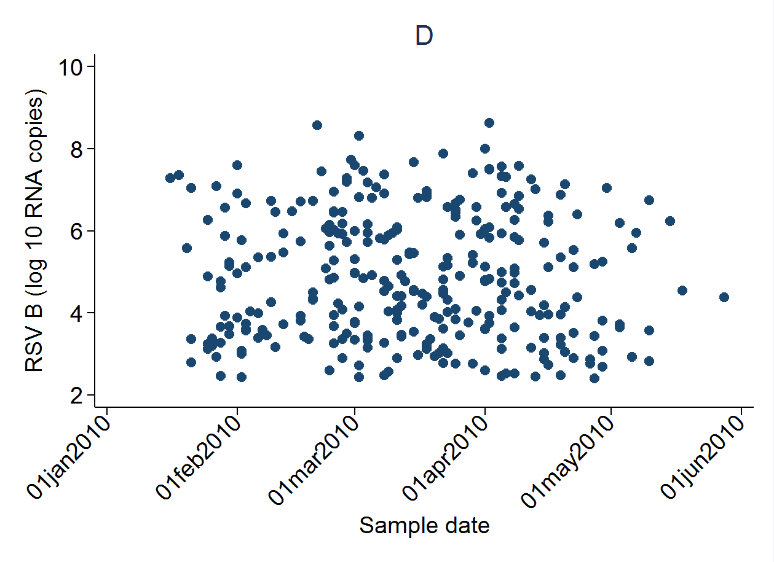

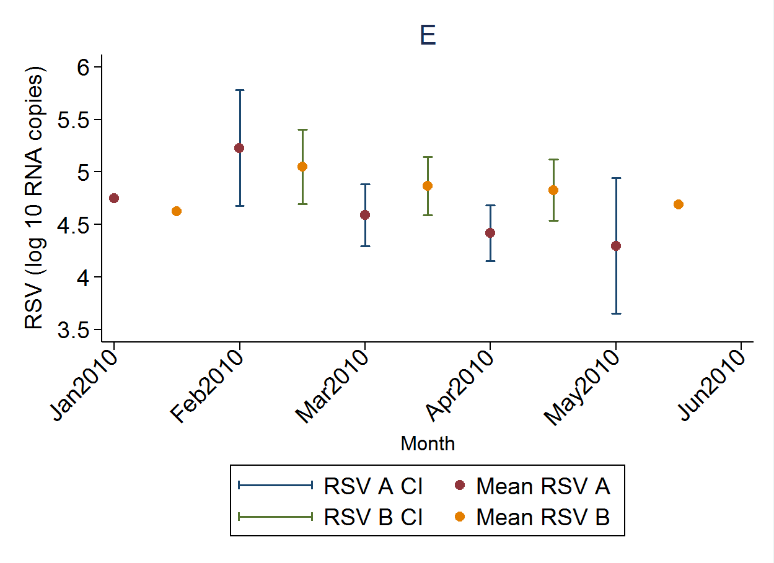


# *Figure S2: Plots showing: a) Ct values for RSV A and b) for RSV B by month with cut off at 35Ct showing that Ct values above 35 or equal to 0 were considered negative c) Converted viral load values for samples positive for RSV A. d) Converted viral load values for samples positive for RSV B. e) Mean and 95% CI for RSV A and B viral load by month for samples positive for RSV.*


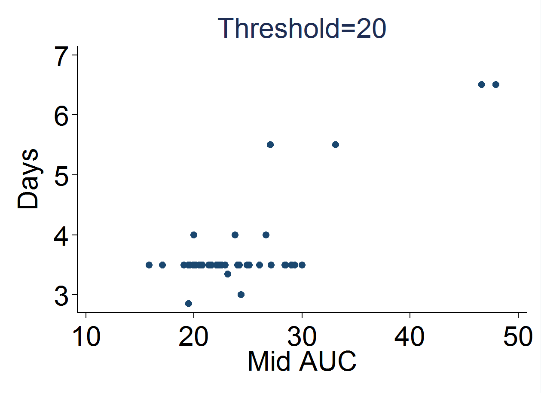

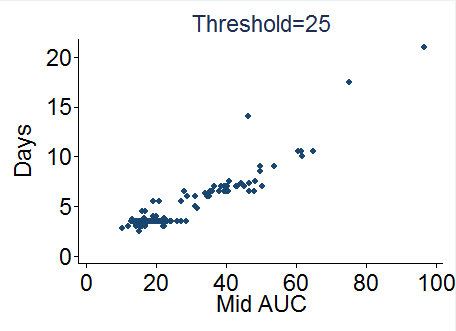

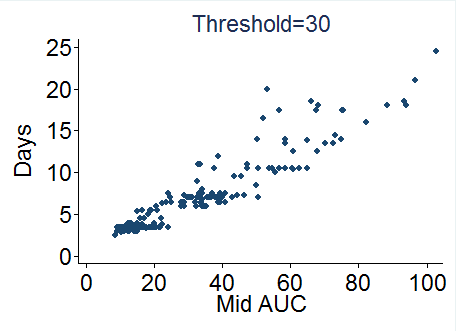


# *Figure S3: Scatter plots showing relationship between amount of virus and duration of shedding for different thresholds of positivity (i.e. Ct=20; Ct=25; Ct=30)*

Table S1: Descriptive analysis: Minimum, midpoint and maximum amount of virus shed by various characteristics

|  |  |  | **Minimum** |  | **Midpoint** |  | **Maximum** |  |
| --- | --- | --- | --- | --- | --- | --- | --- | --- |
| **Characteristic** | **Categories** | **n** | **Median (IQR)** | **P-value** | **Median (IQR)** | **P-value** | **Median (IQR)** | **P-value** |
| Age at infection (years) | <1y | 35 | 58.8 (32.0 – 77.2) | 0.0001 | 71.0 (42.3 - 96.7) | 0.0001 | 79.0 (47.8 - 112.2) | 0.0001 |
|  | 1-<5y | 51 | 25.0 (17.1 - 46.9) | | 37.7 (24.6 – 54.4) | | 43.0 (31.4 - 60.9) |  |
|  | 5-<15y | 73 | 17.8 (2.9 – 28.6) |  | 25.0 (14.1 - 36.7) | | 32.9 (21.8 – 49.3) |  |
|  | 15-<40y | 40 | 2.9 (2.1 - 22.9) |  | 14.6 (9.4 – 32.4) |  | 24.4 (15.1 – 41.1) |  |
|  | > = 40y | 9 | 55.6 (1.3 - 58.4) |  | 56.3 (5.7 – 65.3) |  | 59.3 (12.2 - 67.8) |  |
| Sex | Female | 118 | 19.5 (2.6 – 44.3) | 0.24 | 28.2 (12.9 - 53.9) | 0.23 | 36.0 (20.3 - 58.8) | 0.15 |
|  | Male | 90 | 23.7 (3.5 – 41.4) |  | 32.1 (19.3 – 55.0) | | 39.3 (28.8 – 73.3) |  |
| Symptomatic episodes | No | 90 | 12.9 (2.2 – 22.2) | <0.001 | 19.0 (9.8 - 29.0) | <0.001 | 27.7 (15.2 – 38.9) | <0.001 |
|  | Yes | 118 | 31.8 (17.5 - 56.5) | | 42.5 (25.1 - 66.0) | | 52.8 (34.8 – 78.5) |  |
| Participant in school | No | 119 | 26.4 (5.1 – 55.6) | 0.003 | 33.9 (18.5 – 61.2) | 0.004 | 42.1 (25.7 - 75.7) | 0.011 |
|  | Yes | 89 | 17.7 (2.7 - 30.6) |  | 25.5 (13.1 - 38.0) | | 35.4 (20.7 – 50.6) |  |
|  |  |  |  |  |  | |  |  |
|  |  |  |  |  |  | |  |  |
| Infecting RSV group | RSV A | 83 | 20.9 (2.8 - 38.9) | 0.001 | 26.4 (15.1 - 52.4) | 0.003 | 36.2 (21.8 – 58.5) | 0.008 |
|  | RSV B | 111 | 19.5 (2.9 – 39.9) |  | 28.8 (13.2 - 48.9) | | 38.6 (21.9 – 58.8) |  |
|  | Co-infection | 14 | 53.4 (38.5 – 74.1) | | 66.1 (42.1 – 75.5) | | 70.2 (46.7 - 86.3) |  |
| Order of infection episode in individuals | First | 179 | 23.7 (3.2 – 46.9) | 0.008 | 32.9 (15.1 - 56.3) | 0.006 | 40.9 (24.0 – 67.8) | 0.006 |
|  | Subsequent | 29 | 13.1 (2.5 - 21.6) |  | 24.0 (10.1 - 27.5) | | 30.3 (15.6 – 37.8) |  |
| Introducer of the infection into household | Index case | 70 | 21.3 (2.6 – 39.2) | 0.62 | 29.4 (12.3 – 46.4) | 0.53 | 37.4 (21.1 – 58.5) | 0.81 |
|  | Others | 138 | 22.1 (3.3 – 46.9) |  | 29.8 (16.5 – 56.8) | | 39.0 (24.0 - 66.3) |  |
| Living with smoker in household | No | 181 | 21.6 (3.1 – 44.3) | 0.44 | 30.8 (15.4 – 55.0) | 0.24 | 38.1 (24.0 – 64.3) | 0.32 |
|  | Yes | 27 | 17.9 (2.6 - 38.9) |  | 28.8 (11.5 – 44.5) | | 39.1 (17.9 - 49.3) |  |
| Household size | < = 5 | 25 | 17.8 (2.4 – 58.4) | 0.95 | 30.3 (10.9 - 65.3) | 0.95 | 35.6 (16.9 - 67.8) | 0.94 |
|  | 6 to 10 | 61 | 28.6 (2.7 – 46.7) |  | 36.7 (12.9 - 54.7) | | 42.9 (22.7 – 68.8) |  |
|  | 11 to 15 | 40 | 21.3 (3.1 – 40.7) |  | 28.9 (16.6 - 47.2) | | 38.6 (22.7 – 56.4) |  |
|  | >15 | 82 | 21.3 (3.4 – 36.7) |  | 27.7 (17.7 - 53.7) | | 35.5 (27.4 – 61.2) |  |
| Infection with other viruses |  |  |  |  |  | |  |  |
|  | No | 98 | 17.9 (2.5 - 28.6) | 0.0002 | 25.0 (11.1 – 37.0) | 0.0001 | 34.2 (17.9 – 46.7) | 0.0002 |
|  | Yes | 110 | 29.1 (12.0 – 56.0) | | 38.7 (19.6 – 65.3) | | 49.7 (26.1 – 78.4) |  |
| During household outbreak | No | 16 | 13.1 (2.5 – 25.1) | 0.083 | 20.3 (10.2 – 35.4) | 0.069 | 31.0 (16.4 – 40.9) | 0.096 |
|  | Yes | 192 | 22.6 (3.1 – 45.6) |  | 31.6 (15.9 – 54.8) | | 39.3 (23.3 – 64.1) |  |

Table S2: Descriptive analysis: Amount of virus, Peak viral density and duration of shedding by various characteristics

| **Characteristic** | **Categories** | **n** | **Amount of virus (AUC)** | | **Peak viral load** | | **Duration of shedding** | |
| --- | --- | --- | --- | --- | --- | --- | --- | --- |
|  |  |  | **Median (IQR)** | **P-value** | **Median(IQR)** | **P-value** | **Median(IQR)** | **P-value** |
| Age at infection (years) | <1y | 35 | 71.0 (42.3 - 96.7) | 0.0001 | 7.0 (6.0 – 7.4) | 0.0001 | 15.5 (9.5 – 24.5) | 0.0001 |
|  | 1-<5y | 51 | 37.7 (24.6 – 54.4) |  | 6.3 (5.3 – 6.9) |  | 10.0 (7.0 – 13.0) |  |
|  | 5-<15y | 73 | 25.0 (14.1 - 36.7) |  | 5.2 (4.4 – 6.2) |  | 7.0 (4.0 – 10.5) |  |
|  | 15-<40y | 40 | 14.6 (9.4 – 32.4) |  | 4.8 (3.8 – 6.0) |  | 5.0 (3.5 – 7.5) |  |
|  | > = 40y | 9 | 56.3 (5.7 – 65.3) |  | 5.2 (2.5 – 6.8) |  | 14.0 (3.5 – 14.3) |  |
| Sex | Female | 118 | 28.2 (12.9 - 53.9) | 0.23 | 5.6 (4.1 – 6.7) | 0.06 | 7.0 (4.0 – 13.5) | 0.43 |
|  | Male | 90 | 32.1 (19.3 – 55.0) |  | 5.9 (4.8 – 6.9) |  | 7.5 (5.5 – 14.0) |  |
| Symptomatic episodes | No | 90 | 19.0 (9.8 - 29.0) | <0.001 | 4.8 (3.8 – 5.7) | <0.001 | 6.0 (3.5 – 8.0) | <0.001 |
|  | Yes | 118 | 42.5 (25.1 - 66.0) |  | 6.2 (5.1 – 7.0) |  | 10.5 (6.8 – 17.5) |  |
| Participant in school | No | 119 | 33.9 (18.5 – 61.2) | 0.004 | 6.0 (4.7 – 7.0) | 0.003 | 9.0 (6.0 – 14.3) | 0.035 |
|  | Yes | 89 | 25.5 (13.1 - 38.0) |  | 5.3 (4.3 – 6.3) |  | 7.0 (4.0 – 10.5) |  |
|  |  |  |  |  |  |  |  |  |
|  |  |  |  |  |  |  |  |  |
| Infecting RSV group | RSV A | 83 | 26.4 (15.1 - 52.4) | 0.003 | 5.2 (4.1 – 6.3) | 0.0001 | 7.0 (5.3 – 13.3) | 0.03 |
|  | RSV B | 111 | 28.8 (13.2 - 48.9) |  | 5.9 (4.6 – 6.8) |  | 7.0 (4.0 – 13.0) |  |
|  | Co-infection | 14 | 66.1 (42.1 – 75.5) |  | 7.1 (6.8 – 7.5) |  | 14.0 (10.5 – 18.0) | |
| Order of infection episode in individuals | First | 179 | 32.9 (15.1 - 56.3) | 0.006 | 5.9 (4.7 – 6.9) | 0.0002 | 7.5 (5.0 – 14.0) | 0.11 |
|  | Subsequent | 29 | 24.0 (10.1 - 27.5) |  | 4.8 (3.5 – 5.4) |  | 7.0 (3.7 – 10.5) |  |
| Introducer of infection in household | Index case | 70 | 29.4 (12.3 – 46.4) | 0.53 | 5.9 (4.5 – 6.7) | 0.86 | 7.0 (4.5 – 11.0) | 0.42 |
|  | Others | 138 | 29.8 (16.5 – 56.8) |  | 5.7 (4.5 – 6.7) |  | 7.3 (4.5 – 14.0) |  |
| Living with smoker in household | No | 181 | 30.8 (15.4 – 55.0) | 0.24 | 5.7 (4.5 – 6.7) | 0.2 | 7.5 (5.0 – 14.0) | 0.18 |
|  | Yes | 27 | 28.8 (11.5 – 44.5) |  | 6.2 (4.4 – 7.0) |  | 6.5 (3.8 – 10.5) |  |
| Household size | < = 5 | 25 | 30.3 (10.9 - 65.3) | 0.95 | 5.9 (4.7 – 6.9) | 0.37 | 7.0 (3.5 – 14.0) | 0.73 |
|  | 6 to 10 | 61 | 36.7 (12.9 - 54.7) |  | 5.4 (4.3 – 6.7) |  | 10.5 (4.0 – 14.0) |  |
|  | 11 to 15 | 40 | 28.9 (16.6 - 47.2) |  | 5.3 (4.2 – 6.5) |  | 7.5 (4.0 – 10.8) |  |
|  | >15 | 82 | 27.7 (17.7 - 53.7) |  | 6.0 (4.9 – 6.7) |  | 7.0 (5.5 – 13.5) |  |
| Infection with other viruses | No | 98 | 25.0 (11.1 – 37.0) | 0.0001 | 5.4 (4.4 – 6.2) | 0.0024 | 7.0 (3.5 – 10.5) | 0.0001 |
|  | Yes | 110 | 38.7 (19.6 – 65.3) |  | 6.2 (4.8 – 7.0) |  | 10.5 (6.5 – 16.0) |  |
|  |  |  |  |  |  |  |  |  |
| During household outbreak | No | 16 | 20.3 (10.2 – 35.4) | 0.069 | 4.9 (3.8 – 5.7) | 0.049 | 6.8 (3.8 – 9.3) | 0.16 |
|  | Yes | 192 | 31.6 (15.9 – 54.8) |  | 5.9 (4.6 – 6.7) |  | 7.4 (5.0 – 14.0) |  |
