# Supplementary material for: Quantification and determinants of the amount of respiratory syncytial virus (RSV) shed using real time PCR data from a longitudinal household study
Source: Wellcome Open Res. 2017 Mar 13;1:27. Originally published 2016 Dec 14. [Version 2] doi: 10.12688/wellcomeopenres.10284.2 (PMC5218551; doi:10.12688/wellcomeopenres.10284.2)
Supplement: Peer review history

# Peer review: Referee response for version 1

**DOI:** 10.21956/wellcomeopenres.11076.r19771

**Published:** 2017-01-31

**Authors:** John DeVincenzo

**Affiliations:** 1 Department of Pediatrics, University of Tennessee College of Medicine, Memphis, TN, USA

This is a report that adds significantly to the medical literature on the subject. It is from a well-designed study from a longitudinal community household study in rural Kenya from whom collections of respiratory secretions were collected with and without bias with respect to symptomatology. As such, it is a very valuable database to assess many important clinical questions.

 These authors attempt to leverage this impressive dataset to evaluate quantitative virology relationships to clinical and epidemiological endpoints. This is a very valuable goal and they have generated results and analyses that are important, however, there are numerous problems with this approach which deserve to be reviewed, and their conclusions need to be modified in light of these limitations. 

1. **Relatively non-quantitative collection technique:** Unfortunately, because of the nature of the study (outpatient from village homes), the collections themselves had to be performed using a swab technique. Swab collections (even if done using a deep swab approach) have been shown to produce specimens that are difficult to use for quantitative virology. Variations in depth of swab acquisition, variations in depth of the transition zone from squamous to respiratory epithelial cells within the nose, reduced sensitivity caused by the small volume collection and the large volume dilution required during the nucleic acid extraction technique, and sample to sample variations of the PCR technique (which can not be overcome by re-running the specimen because of lack of sufficient collection volume) all contribute to creating this relatively non-quantitative collection technique.
2. **Relatively non-quantitative PCR techniques employed:** Molecular quantification of viruses within a sample requires a standard curve to assign a quantitative value based upon a Ct value. One cannot use the Ct value itself without it being read from a standard curve. These standard curves can be created externally from the PCR run itself (less desirable) or can be created internally during the actual run which includes the unknown samples from the patients themselves (more desirable). The creation and design of the standard curves is extremely important in determination of quantification and can be based on whole viral genome from infected cells (thereby controlling for the nucleic acid extraction and purification steps, and controlling for the reverse transcriptase step). Less optimal (but much easier to construct) standard curves can be created that utilize cloned cDNA (easily giving a copy number, but not properly controlling for either the nucleic acid extraction, purification, nor reverse transcription steps). Unfortunately, this study apparently has not constructed standard curves, and they rely solely on generated Ct values to imply quantity. It is, in some cases, acceptable to do so when using a single-plex assay (where relative viral concentrations can fairly be compared to the same technique on different specimens). But this approach is never acceptable to use when multiplexed assays are employed (see below). 
3. **Introduction of technique-driven systemic bias in molecular quantification:** This molecular quantification bias comes in two forms: 
  A) multiplex-PCRs detect viral amplicon differently when there are two molecular targets within the same sample based on competition for ions, primers, and probes, and enzymes within the reaction.
  B) The relative amplification efficiency (and the probe-binding-induced fluorescent signal read out by the PCR machine, will be different depending on the probe and the amplicon size and the relative binding efficiencies of the forward and reverse primers. This makes it impossible to quantitatively assess the quantification of two different viruses (RSV-A and B) within a mixed sample and also makes it impossible to compare one target’s quantity (RSV-A) with another target’s quantity (RSV-B). 
4. **Failure to evaluate infectious viral particles and using non-infectious viral particle counts to make conclusions about infectious viral load**. The authors find and conclude that: “The majority of virus available for transmission during an RSV epidemic appears to arise from individuals in their first year of life and therefore undergoing their first RSV infection episode.” Although I agree with this statement, it is important to realize that from data within this manuscript itself, this statement assumes that infectious viral particles equate to quantitative PCR copy number. This assumption is certainly not true. Infectious virus particles are best quantified by a quantitative culture approach. Factors within the respiratory secretions themselves (such as IgA) have been shown to be associated with RSV culture negativity despite persistent high concentrations of RSV genome continuing to be detected within human respiratory secretions after a documented RSV infection ^1^^,^^2^. Infant’s ability to generate RSV-specific IgA is significantly less than the ability of adults to do so during an RSV infection ^3^^,^^4^. The author’s conclusion that infants likely contribute more than do adults to epidemic spread of RSV infection has been carefully modeled in a recent paper ^5^ in which two key factors; infectious viral particle quantity over time, and quantitative measures of human contact activity during times of infection, were incorporated into the model. The implications of these factors promoting RSV community spread should influence the types of RSV vaccines to be developed and the age ranges targeted by these vaccines, and these different vaccine scenarios have been modeled ^5^.
5. **Questions regarding viral load area under the curve calculations.** Any area is geometrically and mathematically defined by multiplying two linear dimensions together. The same is true for viral load area under the curve (in this paper it is called viral density area under the curve for some reason). Viral load AUC is Viral load (units) multiplied by Time (units). The resulting units of viral load are always containing the unit of time. (For example “log PFUe x days”, or “Copies x hours”). The units of viral load AUC in this paper are incorrect and need to be redefined. When one realizes the mathematical definition of AUC, then it is no surprise that Fig 2a and 2b show a lot of correlation between duration of shedding and AUC (because time (duration) is actually in the denominator of the AUC value). 
6. **Problems of determining co-infection with RSV-A and RSV-B.** Students of RSV need to be very careful in reporting and interpreting data regarding co-infections. This paper is no exception. This paper and others assume that when a multiplexed assay reports that the wavelength of the excited probe is orange and another wavelength of the excited probe is green (I am giving a hypothetical example) that this means that both RSV-A and B are present in the sample. However this may or may not be the case. For example, if the virus is an RSV-A virus, the probe for RSV-A binds relatively tightly to the amplicon through strict base-pair matching, and then cleavage of the reporter molecule from the quencher molecule allows the detection of the amplicon (through inhibition of FRET). However, it is very possible that the RSV-B probe will also bind to the amplicon at a certain number of base pairs, and this can result in the RSV-B reporter being detected (erroneously causing the authors to read that RSV-B is also present in the sample). Because of this problem, this paper’s results, analyses, and conclusions regarding “co-infections” of RSV-A and B need to be taken with caution.
7. **Problems with co-infections with RSV and other respiratory viruses** (Adeno, Rhinoviruses, and Coronaviruses). RSV infection causes massive shedding of respiratory epithelial cells into the airway lumen (which are then collected by the swab technique). Any persistent virus present in those shed cells will therefore be detected at higher frequency during an RSV infection. This is an alternative explanation of the correlation between viral load of RSV and detection of these other infections (Top of Pg. 8). This phenomenon carries much different implications than are assumed to be present by these authors. 
8. **Associations with age and outcome (area under the curve viral load). **The authors do an excellent job of assessing the associations between area under the curve and other factors. After observing the problems with the actual viral load area under the curve measurements themselves, (see points 1,2,3,5 above) it appears that their data shows that the primary host characteristic that affects area under the curve viral load is whether or not children have had a prior RSV infection. This fits with other data from the literature. However, it would be nice to know whether young age within the infant age category was predictive of increased area under the curve viral load. Unfortunately, the data set does not contain data on age in weeks, days, or months within the first year of life. I suspect that younger ages within the infant age range, i.e. the RSV-naïve population, will be related to the area under the curve viral load. Other databases will likely need to be leveraged in order to determine this important question. Although the number of infant infections in this database will likely be small, the authors should attempt to break down the ages within the infant age range in order to try to answer this important question. The infant antiviral functional immune response is likely not fully developed at birth, and RSV can be used to interrogate the ontogeny of this immune response ^6^, which can have major implications on vaccine development.
9. **Associations with other factors and outcome (area under the curve viral load).** The manuscript also indicates that presence of respiratory symptoms, intra-household acquisition of infection, being a first infection episode in the season for an individual, and having a co-infection were associated with increased amounts of RSV shed. We should think about the root causes of these associations so that we can understand principles of RSV infection. I do not know the intricacies of the subject acquisition and identification, but is it possible that intra-household transmission simply is a surrogate marker for earlier swabbing of the patient? We know that viral load peaks in the nose shortly after symptom onset, and that viral load declines thereafter. Therefore, having a first swab earlier would create a subject with a greater viral load AUC. An alternative explanation is that perhaps an inoculum effect is present and that children are inoculated within a household transmission with greater amounts of virus, thus causing greater AUC viral load. Past studies have tried to determine an inoculum effect for RSV and crowding does not seem to be a predictor of viral load ^7^^,^^8^. Likewise, in experimental RSV infection models of adults, there appears to be little effect of inoculum on AUC viral load ^2^. It is likely that RSV inoculum affects whether a person becomes infected, but once that person is infected that inoculum has little effect on viral load, AUC or disease severity. I have addressed the issue of co-infection (and alternative explanations for the observed data) in points 6 and 7 above. 
10. **Waning control of RSV infection with advanced age.** Perhaps the greatest contribution of this paper to the medical literature is the finding that area under the curve viral load begins to rise again in adulthood after the age of 40. This is a fascinating finding, and could result from either immune senescence, or reduced exposure to boosting infections of RSV, or a combination of both.  Whether this adult age-related increase in RSV area under the curve viral load is similar in developed countries will need to be evaluated. 

 

 Overall this paper adds significantly to the medical literature into our understanding of RSV in communities. These authors should be lauded and encouraged in their efforts to extract as much information as possible from carefully constructed clinical trials.

I have read this submission. I believe that I have an appropriate level of expertise to confirm that it is of an acceptable scientific standard, however I have significant reservations, as outlined above.

## References

[1] BaggaBCehelskyJEVaishnawAWilkinsonTMeyersRHarrisonLMRoddamPLWalshEEDeVincenzoJP: Effect of Preexisting Serum and Mucosal Antibody on Experimental Respiratory Syncytial Virus (RSV) Challenge and Infection of Adults. *J Infect Dis*.2015;212(11) : 10.1093/infdis/jiv281 1719-25 10.1093/infdis/jiv281 25977264

[2] DeVincenzoJPWilkinsonTVaishnawACehelskyJMeyersRNochurSHarrisonLMeekingPMannAMoaneEOxfordJPareekRMooreRWalshEStudholmeRDorsettPAlvarezRLambkin-WilliamsR: Viral load drives disease in humans experimentally infected with respiratory syncytial virus. *Am J Respir Crit Care Med*.2010;182(10) : 10.1164/rccm.201002-0221OC 1305-14 10.1164/rccm.201002-0221OC 20622030PMC3001267

[3] MulroyNDeVincenzoJBaggaBMealsEHarrisonLSealyBHurwitzJBenhabiliesLCormierSHijanoDR: Infant mucosal RSV-specific IgA response dynamics during and after infection. *Southern Society for Pediatric Research*.2016;

[4] HijanoD et al.: Neonates fail to mount a mucosal immune response against RSV infection. *St. Jude/PIDS Symposium*.2016;

[5] YaminDJonesFKDeVincenzoJPGertlerSKobilerOTownsendJPGalvaniAP: Vaccination strategies against respiratory syncytial virus. *Proc Natl Acad Sci U S A*.2016;113(46) : 10.1073/pnas.1522597113 13239-13244 10.1073/pnas.1522597113 27799521PMC5135296

[6] MillerCRShahAHarrisonLMealsEHyrneCBaggaBKim-HoehamerYBenhabilesLCormierSDeVincenzoJ: The dynamics of respiratory syncytial virus (RSV) replication reveals age-related functional deficiency and maturation of the infant antiviral immune response. *Southern Society for Pediatric Research*.2016;

[7] DeVincenzoJPEl SaleebyCMBushAJ: Respiratory syncytial virus load predicts disease severity in previously healthy infants. *J Infect Dis*.2005;191(11) : 10.1086/430008 1861-8 10.1086/430008 15871119

[8] El SaleebyCMBushAJHarrisonLMAitkenJADevincenzoJP: Respiratory syncytial virus load, viral dynamics, and disease severity in previously healthy naturally infected children. *J Infect Dis*.2011;204(7) : 10.1093/infdis/jir494 996-1002 10.1093/infdis/jir494 21881113PMC3203391

# Response

**Published:** 2017-03-14

**Authors:** James Nokes

***Responses to John DeVincenzo***

 1. Relatively non-quantitative collection technique: Unfortunately, because of the nature of the study (outpatient from village homes), the collections themselves had to be performed using a swab technique. Swab collections (even if done using a deep swab approach) have been shown to produce specimens that are difficult to use for quantitative virology. Variations in depth of swab acquisition, variations in depth of the transition zone from squamous to respiratory epithelial cells within the nose, reduced sensitivity caused by the small volume collection and the large volume dilution required during the nucleic acid extraction technique, and sample to sample variations of the PCR technique (which can not be overcome by re-running the specimen because of lack of sufficient collection volume) all contribute to creating this relatively non-quantitative collection technique.

***We agree that virus quantification from nasopharyngeal swab collections is imperfect and will result in variation arising from the collection technique. This variation is likely to be random and hence should not alter the patterns with respect to covariables, but make observations more noisy. We hope readers understand the limitations and the fact that there are no better collection alternatives.***

 2. Relatively non-quantitative PCR techniques employed: Molecular quantification of viruses within a sample requires a standard curve to assign a quantitative value based upon a Ct value. One cannot use the Ct value itself without it being read from a standard curve. These standard curves can be created externally from the PCR run itself (less desirable) or can be created internally during the actual run which includes the unknown samples from the patients themselves (more desirable). The creation and design of the standard curves is extremely important in determination of quantification and can be based on whole viral genome from infected cells (thereby controlling for the nucleic acid extraction and purification steps, and controlling for the reverse transcriptase step). Less optimal (but much easier to construct) standard curves can be created that utilize cloned cDNA (easily giving a copy number, but not properly controlling for either the nucleic acid extraction, purification, nor reverse transcription steps). Unfortunately, this study apparently has not constructed standard curves, and they rely solely on generated Ct values to imply quantity. It is, in some cases, acceptable to do so when using a single-plex assay (where relative viral concentrations can fairly be compared to the same technique on different specimens). But this approach is never acceptable to use when multiplexed assays are employed (see below).

***While we do not disagree with the reviewer that more accurate quantification of viral RNA is possible, there is no disputing the negative consistent relationship between starting quantity and cycle threshold. Inter-assay variation was assessed through repeated positive controls which were monitored each run. There is, of course, the underlying high degree of underlying biological variation referred to earlier which diminishes the value of downstream precision in RNA load estimation. Hence we recognise we are using only crude estimates, but these were the data available and they produce results which look epidemiologically sensible.***

 3. Introduction of technique-driven systemic bias in molecular quantification: This molecular quantification bias comes in two forms: 

 A) multiplex-PCRs detect viral amplicon differently when there are two molecular targets within the same sample based on competition for ions, primers, and probes, and enzymes within the reaction.

 B) The relative amplification efficiency (and the probe-binding-induced fluorescent signal read out by the PCR machine, will be different depending on the probe and the amplicon size and the relative binding efficiencies of the forward and reverse primers. This makes it impossible to quantitatively assess the quantification of two different viruses (RSV-A and B) within a mixed sample and also makes it impossible to compare one target’s quantity (RSV-A) with another target’s quantity (RSV-B). 

***The observed increase in AUC for co-infections of RSV A and B relative to single infections is perplexing, so we thank the reviewer for the insights regarding interpretation of multiplex PCR data.***

 4. Failure to evaluate infectious viral particles and using non-infectious viral particle counts to make conclusions about infectious viral load. The authors find and conclude that: “The majority of virus available for transmission during an RSV epidemic appears to arise from individuals in their first year of life and therefore undergoing their first RSV infection episode.” Although I agree with this statement, it is important to realize that from data within this manuscript itself, this statement assumes that infectious viral particles equate to quantitative PCR copy number. This assumption is certainly not true. Infectious virus particles are best quantified by a quantitative culture approach. Factors within the respiratory secretions themselves (such as IgA) have been shown to be associated with RSV culture negativity despite persistent high concentrations of RSV genome continuing to be detected within human respiratory secretions after a documented RSV infection. Infant’s ability to generate RSV-specific IgA is significantly less than the ability of adults to do so during an RSV infection. The author’s conclusion that infants likely contribute more than do adults to epidemic spread of RSV infection has been carefully modeled in a recent paper in which two key factors; infectious viral particle quantity over time, and quantitative measures of human contact activity during times of infection, were incorporated into the model. The implications of these factors promoting RSV community spread should influence the types of RSV vaccines to be developed and the age ranges targeted by these vaccines, and these different vaccine scenarios have been modeled.

***We accept that a limitation of the original study was the absence of virus culture. A caution on inferring virus infectiousness using the PCR quantities is explicit in the paper. While further work will be required to link quantitative PCR copy numbers and infectiousness it is worth noting in a carefully designed human challenge studies viral load dynamics by qPCR mirror that of quantitative culture with qPCR consistently overestimating viral load mainly in the recovery phase. This provides evidence of direct proportional relationship between quantitative culture (a measure infectious virus) and qPCR (measuring both infectious and non-infectious viruses)*** ***(DeVincenzo, Wilkinson et al. 2010)*** ***hence our interpretation albeit with caution.***

 5. Questions regarding viral load area under the curve calculations. Any area is geometrically and mathematically defined by multiplying two linear dimensions together. The same is true for viral load area under the curve (in this paper it is called viral density area under the curve for some reason). Viral load AUC is Viral load (units) multiplied by Time (units). The resulting units of viral load are always containing the unit of time. (For example “log PFUe x days”, or “Copies x hours”). The units of viral load AUC in this paper are incorrect and need to be redefined. When one realizes the mathematical definition of AUC, then it is no surprise that Fig 2a and 2b show a lot of correlation between duration of shedding and AUC (because time (duration) is actually in the denominator of the AUC value).

***Thanks for pointing this out. We have changed the units to log 10 copies x days.***

 6. Problems of determining co-infection with RSV-A and RSV-B. Students of RSV need to be very careful in reporting and interpreting data regarding co-infections. This paper is no exception. This paper and others assume that when a multiplexed assay reports that the wavelength of the excited probe is orange and another wavelength of the excited probe is green (I am giving a hypothetical example) that this means that both RSV-A and B are present in the sample. However this may or may not be the case. For example, if the virus is an RSV-A virus, the probe for RSV-A binds relatively tightly to the amplicon through strict base-pair matching, and then cleavage of the reporter molecule from the quencher molecule allows the detection of the amplicon (through inhibition of FRET). However, it is very possible that the RSV-B probe will also bind to the amplicon at a certain number of base pairs, and this can result in the RSV-B reporter being detected (erroneously causing the authors to read that RSV-B is also present in the sample). Because of this problem, this paper’s results, analyses, and conclusions regarding “co-infections” of RSV-A and B need to be taken with caution.

***Co-detection of RSV A and B were in 19 NPS collections yielding to 14 different infection episodes in 7 households. 4 out of the 7 households had more than one co-infection episode.*** ***While acknowledging the challenges of interpreting co-infection data, the outgoingobservations of clustering of the co-detections by individual and co-infection episodes by households does not point to spurious findings.***

 7. Problems with co-infections with RSV and other respiratory viruses (Adeno, Rhinoviruses, and Coronaviruses). RSV infection causes massive shedding of respiratory epithelial cells into the airway lumen (which are then collected by the swab technique). Any persistent virus present in those shed cells will therefore be detected at higher frequency during an RSV infection. This is an alternative explanation of the correlation between viral load of RSV and detection of these other infections (Top of Pg. 8). This phenomenon carries much different implications than are assumed to be present by these authors.

***The reverse argument is also possible. A higher proportion of shed cells would be infected with RSV. Unless concurrent infections were actually in the very same cells (rather than infecting other cells) then this sloughed material would actually have less co-infection than the remaining intact cells.  In addition, the enhanced sloughing of the epithelial cells would have been prominent in symptomatic infection episodes where secretions will have ‘washed’ a larger surface area and the later is adjusted for in our analyses.***

 8. Associations with age and outcome (area under the curve viral load). The authors do an excellent job of assessing the associations between area under the curve and other factors. After observing the problems with the actual viral load area under the curve measurements themselves, (see points 1,2,3,5 above) it appears that their data shows that the primary host characteristic that affects area under the curve viral load is whether or not children have had a prior RSV infection. This fits with other data from the literature. However, it would be nice to know whether young age within the infant age category was predictive of increased area under the curve viral load. Unfortunately, the data set does not contain data on age in weeks, days, or months within the first year of life. I suspect that younger ages within the infant age range, i.e. the RSV-naïve population, will be related to the area under the curve viral load. Other databases will likely need to be leveraged in order to determine this important question. Although the number of infant infections in this database will likely be small, the authors should attempt to break down the ages within the infant age range in order to try to answer this important question. The infant antiviral functional immune response is likely not fully developed at birth, and RSV can be used to interrogate the ontogeny of this immune response, which can have major implications on vaccine development.

***A very good suggestion though our analyses showed no apparent relationship between age and AUC for infants under 1 year. This is why we did not break down age further for this category.***

 9. Associations with other factors and outcome (area under the curve viral load). The manuscript also indicates that presence of respiratory symptoms, intra-household acquisition of infection, being a first infection episode in the season for an individual, and having a co-infection were associated with increased amounts of RSV shed. We should think about the root causes of these associations so that we can understand principles of RSV infection.I do not know the intricacies of the subject acquisition and identification, but is it possible that intra-household transmission simply is a surrogate marker for earlier swabbing of the patient? We know that viral load peaks in the nose shortly after symptom onset, and that viral load declines thereafter. Therefore, having a first swab earlier would create a subject with a greater viral load AUC. An alternative explanation is that perhaps an inoculum effect is present and that children are inoculated within a household transmission with greater amounts of virus, thus causing greater AUC viral load. Past studies have tried to determine an inoculum effect for RSV and crowding does not seem to be a predictor of viral load. Likewise, in experimental RSV infection models of adults, there appears to be little effect of inoculum on AUC viral load. It is likely that RSV inoculum affects whether a person becomes infected, but once that person is infected that inoculum has little effect on viral load, AUC or disease severity. I have addressed the issue of co-infection (and alternative explanations for the observed data) in points 6 and 7 above. 

***Swabs were collected regardless of symptom status and days of collections were planned to coincide for all members in same household unless someone was away. Therefore swabbing in early phase of infection (when viral load was high) would have occurred by chance and would influence the observed associations towards null.***

 10. Waning control of RSV infection with advanced age. Perhaps the greatest contribution of this paper to the medical literature is the finding that area under the curve viral load begins to rise again in adulthood after the age of 40. This is a fascinating finding, and could result from either immune senescence, or reduced exposure to boosting infections of RSV, or a combination of both.  Whether this adult age-related increase in RSV area under the curve viral load is similar in developed countries will need to be evaluated.

***Indeed this is an intriguing observation. We have interpreted this finding with caution since we had rather small sample size for these age group i.e. 9 episodes from 8 infected participants.***

 DeVincenzo, J. P., T. Wilkinson, A. Vaishnaw, J. Cehelsky, R. Meyers, S. Nochur, L. Harrison, P. Meeking, A. Mann, E. Moane, J. Oxford, R. Pareek, R. Moore, E. Walsh, R. Studholme, P. Dorsett, R. Alvarez and R. Lambkin-Williams (2010). "Viral load drives disease in humans experimentally infected with respiratory syncytial virus." Am J Respir Crit Care Med 182(10): 1305-1314.

# Peer review: Referee response for version 1

**DOI:** 10.21956/wellcomeopenres.11076.r18492

**Published:** 2017-01-03

**Authors:** Peter F. Wright

**Affiliations:** 1 Department of Pediatrics, Geisel School of Medicine, Dartmouth College, Hanover, NH, USA

The report by the KEMRI team builds on a cohort of 47 households followed through an RSV season in coastal Kenya with frequent nasopharyngeal swabs for detection of RSV. The introduction and rationale for the study are very clearly provided.

 More complicated is the analysis to determine minimum and maximum areas under the curve representing estimates of the total amount of virus shed based on a PCR cycle to detection of virus. Once this is accepted the analysis of factors influencing the amount of virus detected is straightforward and indicates age, symptoms, RSV type B, and whether infection is the initial infection during the sampling period. Each of these observations remained significant in a multiple linear regression analysis. None of these observations are surprising but all are of interest in this locale and within a community based study design. Might the large number of asymptomatic individuals include some who subsequently became symptomatic and were at the time of sampling in the incubation period of their illness?

 The authors address some limitations of the study. Other things that might be considered include an analysis based gradation of symptoms in terms of virus recovered, whether frequency of sampling was influenced by symptoms, and patterns of spread within families. The latter is of particular interest in that I suspect that with many viral infections the peak titre will influence spread more than the total amount shed. The number of days to detection of secondary infections in the household might give a clue to the validity of this hypothesis.

I have read this submission. I believe that I have an appropriate level of expertise to confirm that it is of an acceptable scientific standard.

# Response

**Published:** 2017-01-25

**Authors:** Miriam Wathuo

**Affiliations:** KEMRI Wellcome Trust Research Programme, Kenya

1. Might the large number of asymptomatic individuals include some who subsequently became symptomatic and were at the time of sampling in the incubation period of their illness? 

***Being symptomatic or not was defined both by individuals and by episodes.***

***An episode was described as asymptomatic if the individual never had any respiratory illness symptoms during the entire duration of the infection episode. An episode was described as symptomatic if the individual had respiratory illness symptoms at least once during the episode.***

***An individual was described as symptomatic if they had respiratory illness symptoms at least once during the study period, and asymptomatic if they never had any respiratory illness symptoms throughout the study period.***

***Of the 71 individuals with asymptomatic episodes, 19 (26.8%) subsequently developed symptoms within one week. The subsequent symptoms were not linked to any virus detection in 15 (78.9%) of the 19 individuals.***

 2. Other things that might be considered include an analysis based gradation of symptoms in terms of virus recovered, whether frequency of sampling was influenced by symptoms, and patterns of spread within families.

***Sampling was done irrespective of symptom status and the frequency of sampling was not influenced by symptoms. Mild symptoms (mainly cough and running nose) were observed among the symptomatic cases hence difficult to grade the disease severity – no severe or very severe pneumonia cases. Nevertheless, there was no association between symptom status and virus type. The patterns of spread within households and role of asymptomatic cases has been discussed in detail in previous papers: ***

1. ***Munywoki PK, Koech DC, Agoti CN, et al. The source of respiratory syncytial virus infection in infants: a household cohort study in rural Kenya. [electronic article]. 2014;209(11):1685–92.***
2. ***Munywoki, P. K., D. C. Koech, C. N. Agoti, A. Bett, P. A. Cane, G. F. Medley and D. J. Nokes (2015). "Frequent Asymptomatic Respiratory Syncytial Virus Infections During an Epidemic in a Rural Kenyan Household Cohort." J Infect Dis 212(11): 1711-1718.***
3. ***Munywoki, P. K., D. C. Koech, C. N. Agoti, N. Kibirige, J. Kipkoech, P. A. Cane, G. F. Medley and D. J. Nokes (2015). "Influence of age, severity of infection, and co-infection on the duration of respiratory syncytial virus (RSV) shedding." Epidemiol Infect 143(4): 804-812.***

# Peer review: Referee response for version 1

**DOI:** 10.21956/wellcomeopenres.11076.r18488

**Published:** 2016-12-30

**Authors:** H. Rogier van Doorn, Lien Anh Ha Do

**Affiliations:** 1 Oxford University Clinical Research Unit- Hanoi, Wellcome Trust Major Overseas Programme, National Hospital of Tropical Diseases, Hanoi, Vietnam; 2 Murdoch Children’s Research Institute, Melbourne, VIC, Australia

This is an interesting paper using area under curve (AUC), derived from non-quantitative PCR data using three different estimates, as a unit to measure viral shedding during an RSV season 8 years ago for a prospective cohort of 47 households in rural coastal Kenya. AUC can take variability of viral load due to different factors (technical, biological) into account and reflects the overall temporal changes of virus shed.

 The authors use three models for calculating the AUC. While this is not my expertise, I cannot comment on these models, but I strongly believe that choosing an appropriate model is one of the most crucial parts of this study, any wrong assumption/ formula of these models will affect the results and its interpretations. Hence, the introduction of AUC as a measurement unit for transmission studies is an interesting concept, however, it may need a validation step using data from animal models, human challenge models, or clinical data monitoring RSV load.

 Below are some points requiring more clarification:

1. The interval between samples is 3-4 day, why did the author choose this big interval, while previous human challenge studies have suggested daily sampling or every 2 days could be the best follow-up (1). This is a major limitation as samples are taken only every 3-4 days and amounts of shed virus are therefore modelled based on little data and many assumptions, which may or may not equal out. The authors should definitely include more information on how their semi-quantitative data was calibrated.
2. As authors didn’t use qPCR and use of a standard curve is not described, authors should clarify how they ensured PCR efficiency was stable between runs and what was used to calibrate runs and Ct values. This is a crucial thing to add, as all results in this paper are based on (semi)quantitative measurements and therefore, proper quantification is absolutely key.
3. At the beginning of the Methods section, the authors specified that the AUC was calculated using viral load in log scale, but later for the midpoint AUC, they used Ct-value, please clarify.
4. Reference 21 and 23 are not accessible
5. Without baseline demographics about the sampled cohort I find some of this information hard to interpret. Symptomatic infections would also be interesting to see by age group.
6. In Table 1, it is not clear how the total AUC was calculated. Surprisingly, the number of RSV infection episodes in the young age group (less than 1 year old and less than 5 years old) is much lower (denominator?) than those in the age group of 5-15 years old, while many epidemiological data have shown the re-infection rates are higher in the young age group. There is no comment/discussion on this result.
  In addition, the number of RSV infection episodes and the AUC in “living with smokers” group were lower and similar, respectively, compared to those who do not live with smokers. This result is controversial with the common observations from many epidemiological studies on RSV susceptibility and there is no comment/discussion regarding this.

Specific minor comments: 

1. P3c1p2: The comparison with human volunteer studies to suggest load / inoculum may be associated with infection is not very strong, this may only be true in the background of a well-developed immune history against RSV whereas the main burden of disease of RSV is in infants who will not have this history yet.
2. P3c1p3: I don’t think excretion is proper terminology for the process of virus shedding (nor is viral density – which is used later on, p6).
3. P3c1p4: the number of sampled household members and the inclusion criteria should be added here.
4. P3c2p1: why were only adeno, corona and rhino targeted with the diagnostic PCR and not the influenzas, parainfluenzas and hMPV? This is a very limited definition of co-infection.         
    
5. P6: Please clarify why data are presented as median (IQR) amounts of virus shed in one paragraph but then as mean amounts (95%CI) of virus shed in the other.
6. P8c1p2: In Table 2 sex is not significant, but its mentioned here as an associated factor.
7. P8c2p3: the result that timing within the season was (appeared?) associated with the amount of virus shed is presented in the discussion section, shouldn’t this also be presented as a result, with data and a p-value?
8. P8c2p3: why is it of particular importance that 90.3% of virus shed is from individuals experiencing their first episode? Also, 179/208 episodes studied were first episodes.
9. Table 2: the number of primary and secondary cases per age group would be informative here

In summary, this paper reports important data on RSV transmission in a community setting; data on this subject is still scarce. This data is relevant for RSV vaccine and treatment development and can help suggest target groups for vaccination and implications of infection control measurement.

We have read this submission. We believe that we have an appropriate level of expertise to confirm that it is of an acceptable scientific standard, however we have significant reservations, as outlined above.

## References

[1] DeVincenzoJPWilkinsonTVaishnawACehelskyJMeyersRNochurSHarrisonLMeekingPMannAMoaneEOxfordJPareekRMooreRWalshEStudholmeRDorsettPAlvarezRLambkin-WilliamsR: Viral load drives disease in humans experimentally infected with respiratory syncytial virus. *Am J Respir Crit Care Med*.2010;182(10) : 10.1164/rccm.201002-0221OC 1305-14 10.1164/rccm.201002-0221OC 20622030PMC3001267

# Response

**Published:** 2017-01-25

**Authors:** Miriam Wathuo

**Affiliations:** KEMRI Wellcome Trust Research Programme, Kenya

1. The interval between samples is 3-4 day, why did the author choose this big interval, while previous human challenge studies have suggested daily sampling or every 2 days could be the best follow-up (1). This is a major limitation as samples are taken only every 3-4 days and amounts of shed virus are therefore modelled based on little data and many assumptions, which may or may not equal out. The authors should definitely include more information on how their semi-quantitative data was calibrated.

***We agree the sampling interval was not optimal for this type of analysis. However, it was not logistically possible to collect deep nasopharyngeal swabs from ~500 participants at a more intense frequency for six months. Furthermore, community acceptability of more frequent sampling was an important issue.***

***The following rationale was used to determine the twice-weekly (every 3.5 days) nasal sampling frequency. Assuming individuals shed virus with mean duration of between 3.5 and 9 days, with a constant rate of recovery from shedding, and an onset on average half way between any sampling interval, then the proportion of individuals predicted to remain shedding, and thus detectable, will range from 61%-82% (for 3.5-9 days duration) for a 3.5 day sampling interval. Given the need to detect infection in mild cases and in older children and adults with a likely lower range of shedding duration, sampling twice weekly was indicated.***

 2. As authors didn’t use qPCR and use of a standard curve is not described, authors should clarify how they ensured PCR efficiency was stable between runs and what was used to calibrate runs and Ct values. This is a crucial thing to add, as all results in this paper are based on (semi) quantitative measurements and therefore, proper quantification is absolutely key.

***This is a retrospective analysis of household RSV infection data and we share the referee’s concerns about limitations on the use of Ct values. A qPCR would have been the best method to quantify viral load. However, a standardized multiplex RT-PCR protocol was used which included positive controls monitored each run to ensure their Ct values did not vary significantly.***

 3. At the beginning of the Methods section, the authors specified that the AUC was calculated using viral load in log scale, but later for the midpoint AUC, they used Ct-value, please clarify.

***Viral load for calculation of minimum, midpoint and maximum AUC was presented in log _10_ copies. Ct values are mentioned to explain how viral load for days in between collection days was estimated before being converted to log _10_ copies. The original viral load values were in Ct values, which were then converted to log _10_ copies and used to calculate AUC in all three scenarios (minimum, midpoint and maximum AUC).*** ***Please also see comments in response to Referee Cowling.***

 4. Reference 21 and 23 are not accessible

***Thank you. We have rectified the errors in the two references. Reference 21 should be:***

***‘*** ***Kreide, Donald; Lahr D. Trapezoid Rule and Simpson’s Rule [Internet]. 2010. Available from:*** ***https://math.dartmouth.edu/~m3cod/klbookLectures/406unit/trap.pdf*** ***’,***

******

***while reference 23 is the study dataset.***

 5. Without baseline demographics about the sampled cohort, I find some of this information hard to interpret. Symptomatic infections would also be interesting to see by age group.

***We have provided a brief summary of the baseline characteristics in the first paragraph of the results section. However, more details could be accessed in our earlier publications arising from this study whose references are provided. Figure 4 of this manuscript provides distribution of symptomatic and asymptomatic infections by age.***

 6.  In Table 1, it is not clear how the total AUC was calculated. Surprisingly, the number of RSV infection episodes in the young age group (less than 1 year old and less than 5 years old) is much lower (denominator?) than those in the age group of 5-15 years old, while many epidemiological data have shown the re-infection rates are higher in the young age group. There is no comment/discussion on this result.

***Total AUC is the cumulative AUC for all infection episodes in a specified category eg age group. The higher number of infection episodes in the 5-15 year age group is most likely due to the higher number of individuals in this age category*** ***, and this translated to the higher total of the amount of virus shed by this age group. To compare the transmission potential per person by age group, an average amount of virus shed per infection episode might be a better statistic rather than the total AUC*** ***. However, from the perspective of contribution to community transmission then the cumulative AUC seems appropriate.***

 7. In addition, the number of RSV infection episodes and the AUC in “living with smokers” group were lower and similar, respectively, compared to those who do not live with smokers. This result is controversial with the common observations from many epidemiological studies on RSV susceptibility and there is no comment/discussion regarding this.

***In this rural community, indoor smoke from solid fuels (mainly firewood and charcoal) was ubiquitous and likely to conceal any effects of smoke from cigarettes. In addition, we only had 18 smokers out of the 493 participants. So, relatively few people lived with smokers. Also, we cannot be sure how much contact members of a household had with a smoker and if the smoker did smoke in the house or outside. A comment has been added in the discussion to that effect.***

 Specific minor comments: 

 1. P3c1p2: The comparison with human volunteer studies to suggest load / inoculum may be associated with infection is not very strong, this may only be true in the background of a well-developed immune history against RSV whereas the main burden of disease of RSV is in infants who will not have this history yet.

***This limitation in extrapolating results on association of infection acquisition and exposure and viral load from human challenge studies has been noted and discussed in the main text.***

 2. P3c1p3: I don’t think excretion is proper terminology for the process of virus shedding (nor is viral density – which is used later on, p6)

***Well noted. We have now used viral shedding consistently in the text.***

 3. P3c1p4: the number of sampled household members and the inclusion criteria should be added here.

***This has been described in a previous paper as stated in the methods section***

 4. P3c2p1: why were only adeno, corona and rhino targeted with the diagnostic PCR and not the influenzas, parainfluenzas and hMPV? This is a very limited definition of co-infection.

***Adeno, corona and rhino were the common respiratory viruses detected in a preliminary screening of the samples from 5 randomly selected households and taken forward for the screening of rest of the samples from the remaining households.*** ***We thus have limited data to comment on the other respiratory pathogens.***

 5. P6: Please clarify why data are presented as median (IQR) amounts of virus shed in one paragraph but then as mean amounts (95%CI) of virus shed in the other

***The AUC values were still slightly skewed even after log transformation. So, we presented medians in the descriptive analysis because it is the best measure for a skewed variable. However, the linear regression analysis coefficients are means not medians, so for the linear regression results, we had to present means and 95% CI.***

 6. P8c1p2: In Table 2 sex is not significant, but its mentioned here as an associated factor

***We chose to adjust for sex a priori because sex has previously been considered an important risk factor to RSV infections. In the final model presented sex was not a significant covariate though.***

 7. P8c2p3: the result that timing within the season was (appeared?) associated with the amount of virus shed is presented in the discussion section, shouldn’t this also be presented as a result, with data and a p-value?

***This was a comparison of viral load values (not amount of virus) against time just to show the reader a visual of how viral load varied depending on the month of the year. This was to demonstrate that there is an increase in the number of positive viral load detections during the RSV season. This has been mentioned in the results and discussion sections.***

 8. P8c2p3: why is it of particular importance that 90.3% of virus shed is from individuals experiencing their first episode? Also, 179/208 episodes studied were first episodes

***First episode in this case refers to the first infection detected over the study period, which were 179 (86%).*** ***As stated earlier, the total AUC gives an idea of which groups contribute most to community transmission of virus. It is, therefore, important to note that most virus shed comes from individuals experiencing their first episode of the RSV season.***

 9. Table 2: the number of primary and secondary cases per age group would be informative here

***Thank you. This has been added***.

# Peer review: Referee response for version 1

**DOI:** 10.21956/wellcomeopenres.11076.r18490

**Published:** 2016-12-23

**Authors:** Benjamin J. Cowling

**Affiliations:** 1 WHO Collaborating Centre for Infectious Disease Epidemiology and Control, School of Public Health, Li Ka Shing Faculty of Medicine, The University of Hong Kong (HKU), Hong Kong, China

This is an interesting manuscript about RSV shedding, in the context of household transmission. There are few data on this topic in the literature and this is a valuable contribution. I thought the manuscript was clear and well written and should be indexed. I only had one comment:

1. Please comment on the choice of log viral load in analyses

In Figure 1 please clarify the y-axis metric and you might like to explain this choice as well.

 Is it viral density (copies), or log_10 copies ?

 It seems as if you are calculating the AUC based on log_10 viral copies but what is the biological interpretation of that? I was wondering whether the number of viruses could be a better correlate of transmission, not their log?

I have read this submission. I believe that I have an appropriate level of expertise to confirm that it is of an acceptable scientific standard.

# Response

**Published:** 2017-01-25

**Authors:** Miriam Wathuo

**Affiliations:** KEMRI Wellcome Trust Research Programme, Kenya

1. Please comment on the choice of log viral load in analyses

***The absolute values (viral RNA copy numbers) for AUC were not normally distributed and were log transformed to stabilize the variances providing a suitable outcome variable for use in linear regression analysis. The mean (SD) for the untransformed and log-transformed values is 3.73x10 ^7 ^(1.34x10 ^8^) and 6.07 (1.51), respectively. The choice of outcome has been clarified in the manuscript.***

***We have re-analysed the data by the alternative route of summing the absolute viral load values and then taking logs to estimate the AUC. The results are not substantially different from the current method which is a summation of the log-transformed values to estimate AUC. This is stated in the discussion.***

 2. ****In Figure 1 please clarify the y-axis metric and you might like to explain this choice as well. Is it viral density (copies), or log_10 copies?

***Log _10_ viral RNA copies as they are the units used in measuring the viral load.***

 3. It seems as if you are calculating the AUC based on log_10 viral copies but what is the biological interpretation of that? I was wondering whether the number of viruses could be a better correlate of transmission, not their log? 

***The relationship between viral shedding and infectiousness is unknown. The relationship between amount of virus shed and risk of infection might be linear or have a threshold effect (e.g. minimum dose), in which case a logarithmic scale would be more appropriate. The log-transformation allowed us to stabilize the variance of the outcome values, suggesting that the measurement error of amount of virus might vary non-linearly. The final c*** ***onclusions (biological interpretation of determinants of amount of virus spread) is similar to when using the arithmetic values since an increase in log values is also linked to an increase in absolute values albeit at exponential scale.*** 

***The relationship between various factors such as age, symptoms, etc and untransformed amount of virus is the same as that between those factors and the log-transformed amount of virus. Using log-transformed values does not affect the final conclusions. Determining the relationship between measures of virus shed and risk of transmission requires a different analysis which we are undertaking. This is now reflected in the Discussion.***
